# EEG correlates of auditory rise time processing: A systematic review

**DOI:** 10.1371/journal.pone.0345343

**Published:** 2026-10-06

**Authors:** Victoria Manasevich, Daria Kostanian, Anton Rogachev, Olga Sysoeva

**Affiliations:** 1 Human Development: Brain and Mind, Centre for Research on Talent Development, Sirius University of Science and Technology, Federal Territory Sirius, Russia; 2 Laboratory of Human Higher Nervous Activity, Institute of Higher Nervous Activity and Neurophysiology, RAS, Moscow, Russia; Nathan S Kline Institute, UNITED STATES OF AMERICA

## Abstract

Rise time (RT) is a critical acoustic feature of speech, implicated in typical and atypical language development. While a substantial amount of data has been accumulated on the neurophysiological evidence of RT processing across different conditions and groups of people, these data have not been systematized. This systematic review, conducted according to the PRISMA guidelines across PubMed, Web of Science and APA PsychInfo databases, focuses on studies that have investigated electroencephalographic (EEG) markers of RT sensitivity in humans. A total of 37 studies were identified that manipulated RT and examined different components of event-related potentials (ERPs), brainstem responses, and cortical rhythmic activity. The core finding is consistent across studies: RT prolongation reduces the amplitudes of brain responses and increases their latencies. However, the sensitivity of the EEG markers varied along the auditory hierarchy with the earliest components tracking subtle differences (a few tens of microseconds), while the later components coding the larger changes (up to 500 ms). Effects are further modulated by the experimental parameters, participant age, and speech-related problems, while limited pediatric research, considerable methodological heterogeneity and predominantly small sample sizes limit the clear conclusions on these modulations. Future research should prioritize understudied clinical populations and developmental ERP components such as P1 and N2, which are dominant in children.

## Introduction

The ability to process the acoustical characteristics of auditory speech stimuli is an important predictor of speech development [1]. One such characteristic is auditory rise time (RT) – the time taken for the amplitude of a sound to rise from a low to a high value, effectively describing how sharply or gradually a sound’s envelope reaches its peak [2]. Perceptually, a shorter RT creates a more abrupt onset, whereas a longer RT produces a smoother, more gradual onset. Very short RTs (<10 ms) produce spectral splatter, which is perceived as a click.

In natural speech, RT cues are critical for parsing the continuous acoustic stream into discrete phonological units. For example, the distinction between the stop consonant /b/ and the glide /w/ relies partly on the rate of amplitude rise at sound onset: rapid amplitude increase produces abrupt stop/click, whereas slower rise is perceived as a glide [3]. Similarly, the distinction between the voiceless affricate /tʃ/ and the fricative /ʃ/ also involves differences in the amplitude envelope’s rise characteristics, although the perceptual boundary between these categories is not fixed but shifts depending on the stimulus context [4]. Thus, amplitude RT contributes directly to phonetic categorization across multiple types of consonant contrasts. It should be noted that RT is sometimes distinguished into amplitude RT and formant RT – the rate at which formant frequencies reach their target values; the two are often discussed together, but the present review focuses on the amplitude rise time specifically.

Accurate RT perception is thought to support the formation of stable phonological representations, which in turn are essential for mapping sounds to letters during reading acquisition [1, 2]. Psychophysical studies of RT sensitivity provide a framework for assessing the contribution of phonological processing to speech and language development. The difference threshold (i.e., the minimal change in RT at which stimuli are perceived as different) is used to estimate the measure of RT sensitivity. Individual differences in the ability to discriminate RTs may affect the accuracy of linguistic representations, which may be related to individual variation in speech performance: less reliable encoding of RT cues may weaken the distinctiveness of phonological categories to which they contribute, leading to less stable phonological categories [1]. Children with dyslexia have demonstrated less sensitivity to amplitude RT of auditory stimuli than their control group peers [1]. In addition, sensitivity to RT in infancy may act as a predictor of vocabulary development at an older age [2].

At the neurophysiological level, sensitivity to RT is reflected in the event-related potentials (ERP), a commonly used technique for studying brain responses to stimuli recorded via electroencephalography (EEG). Different ERP components elicited within specific time windows reflect distinct stages of the neurophysiological processing of the stimulus. A large amount of EEG data on RT effects has been accumulated across populations using different methods (ERP, analysis of rhythmic brain activity, etc.), but these data are to some degree inconsistent. This inconsistency, combined with the lack of a comprehensive review encompassing the mechanisms of RT perception from brainstem to cortex, necessitates systematic synthesis. Accordingly, the aim of this systematic review is to evaluate EEG characteristics as markers of RT sensitivity in typically developing children and adults, as well as in groups with different speech disorders. We consider various analytical approaches to capture the full spectrum of RT-related neurophysiological effects.

## Methods

This review followed the methodological framework for systematic reviews. To ensure transparent and complete reporting, we adhered to the PRISMA 2020 guidelines. The PRISMA 2020 checklist has been completed and is available as supplementary material (S1 Table), and the PRISMA flow diagram (Fig 1) illustrates the study selection process. Although a formal review protocol was not previously registered or published, the search strategy, inclusion criteria, and data extraction forms were developed a priori and piloted by the research team to minimize bias.

**Fig 1:**
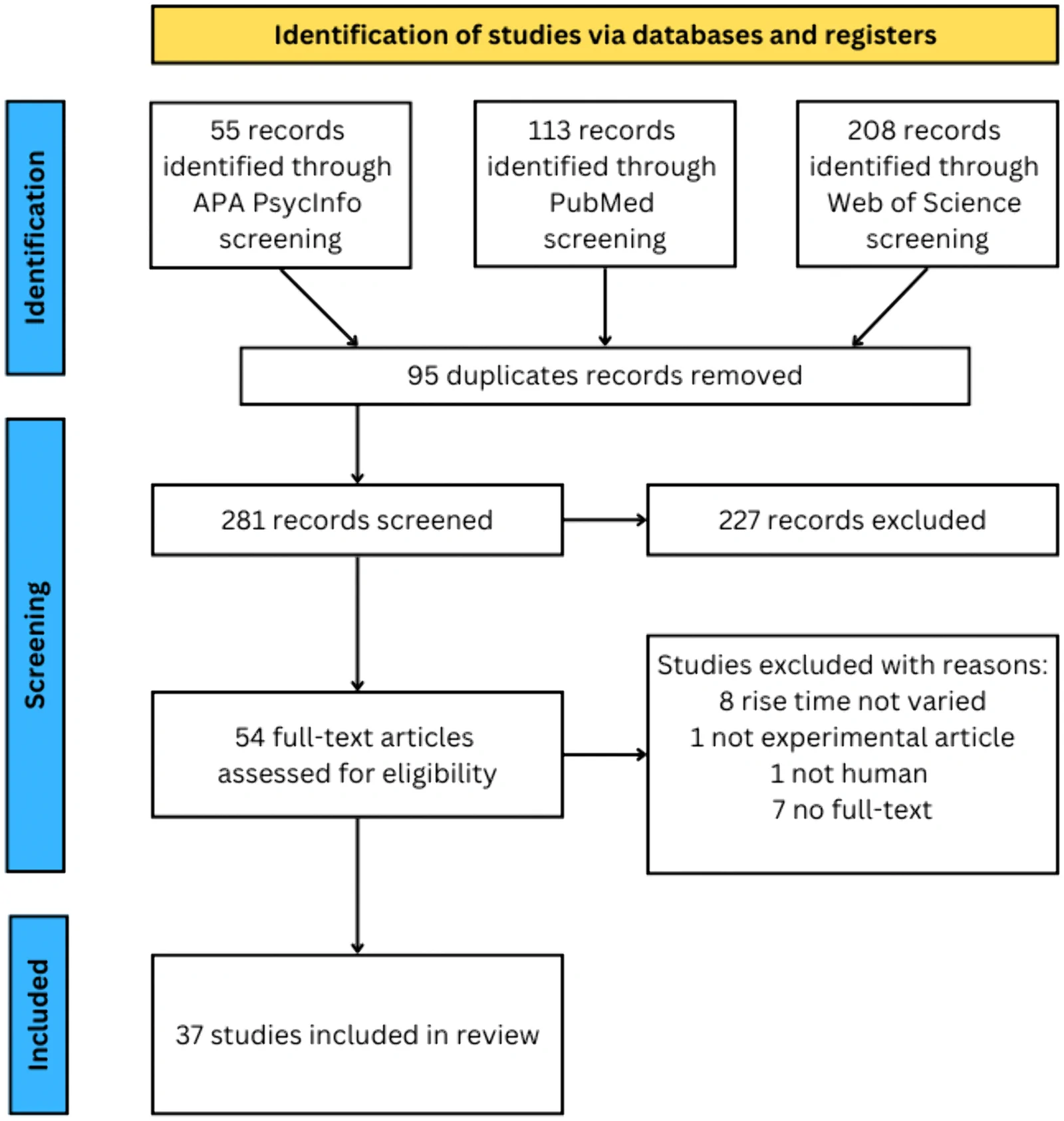
A PRISMA flow-chart representing a flow of information through the different phases of a systematic review.

### Search strategy

We conducted a literature search of the PubMed, Web of Science, and APA PsycInfo databases. The search query used was “(“Rise time”[All Fields] AND (“evoked potentials”[All Fields] OR “erp”[All Fields] OR “evoked response”[All Fields] OR “aep”[All Fields] OR “auditory evoked response”[All Fields] OR “eeg”[All Fields] OR “electrophysiological”[All Fields])) AND (“humans”[Terms] AND “english”[Language]))”. Thus, the search strategy was focused on EEG-based studies, as the majority of systematic investigations of rise-time processing – where rise time is varied as an independent variable – have been conducted using this method. While a small number of magnetoencephalography (MEG) studies have examined rise-time-related phenomena (e.g., sound detection of slowly rising intensity, perceptual asymmetry for ramped vs. damped sounds), they address different research questions and do not systematically vary rise time as a phonetic/acoustic parameter; therefore, they were not included in order to maintain the coherence of the reviewed evidence. We used the specific term “rise time” to target studies directly investigating this acoustic parameter. Terms such as “auditory edges” or “acoustic boundaries” were not included, as they typically refer to studies on general auditory scene segmentation and spectrotemporal boundary detection, which are conceptually distinct from rise time as a parameter of speech signals.

The identified articles were published prior to January 25, 2024. On February 15, 2026, an updated search was performed using the same search strategies and databases as the original search. The search was limited to the period from January 24, 2024, to February 15, 2026, using filters based on entry date. The updated search did not identify any new studies that met the inclusion criteria.

### Eligibility criteria

Only empirical human studies published in English in peer-reviewed journals were included in this review. Articles were retained if they measured ERPs or other EEG data in response to auditory stimuli with varied amplitude/formant RT. Studies were excluded if they contained only one RT condition with no variations.

All titles and abstracts identified in the search were double-blind screened by two independent experts using the SRDR plus platform (https://srdrplus.ahrq.gov) to exclude obviously irrelevant articles. Any disagreements were resolved through discussion with the involvement of a third expert. The records that passed the initial screening were evaluated using their full-text versions. As in the abstract screening, two independent decisions were made for each screened item. In cases of disagreement, the final decision was made after discussion with the involvement of a third expert. The remaining articles were included in the review.

### Data extraction

The following data were extracted onto a custom sheet, which included information about the sample (participant groups, sample size, mean age or age range), the experimental paradigm (oddball, block design or other), stimulus properties (stimulus type, RT conditions, technical parameters of presentation), additional assessments used in the study (e.g., reading skills assessment or an additional deviant condition in an oddball study) and results (reported as EEG parameter changes with RT increase, or observed mismatch negativity (MMN) in response to RT deviants). The extracted data are presented in S2 Table.

The risk of bias was assessed using the OHAT risk of bias tool [5]. The evaluation included three sections containing several questions: selection bias, detection bias, and “other sources of bias” sections. A table with the risk of bias evaluation results is provided in the supplementary materials section (S3 Table).

## Results

A total of 269 articles were identified from all searched databases after duplicate removal. After abstract and full-text screening, 37 articles remained and were included in the review. The summary details of the reviewed studies are presented in S2 Table. Seven articles were excluded from the study due to unavailability of full texts. This was due to the fact that the articles were published a long time ago (1964–1994) and their full texts were not digitized or not available [6–12]. An updated search (February 2026) identified 12 new records, none meeting inclusion criteria at titles and abstracts screening stage. A flowchart of the selection process is shown in Fig 1.

Studies included in the final review ranged in date from 1968 to 2022 (Fig 2), with an evident 11–year gap between 1996 and 2007.

**Fig 2:**
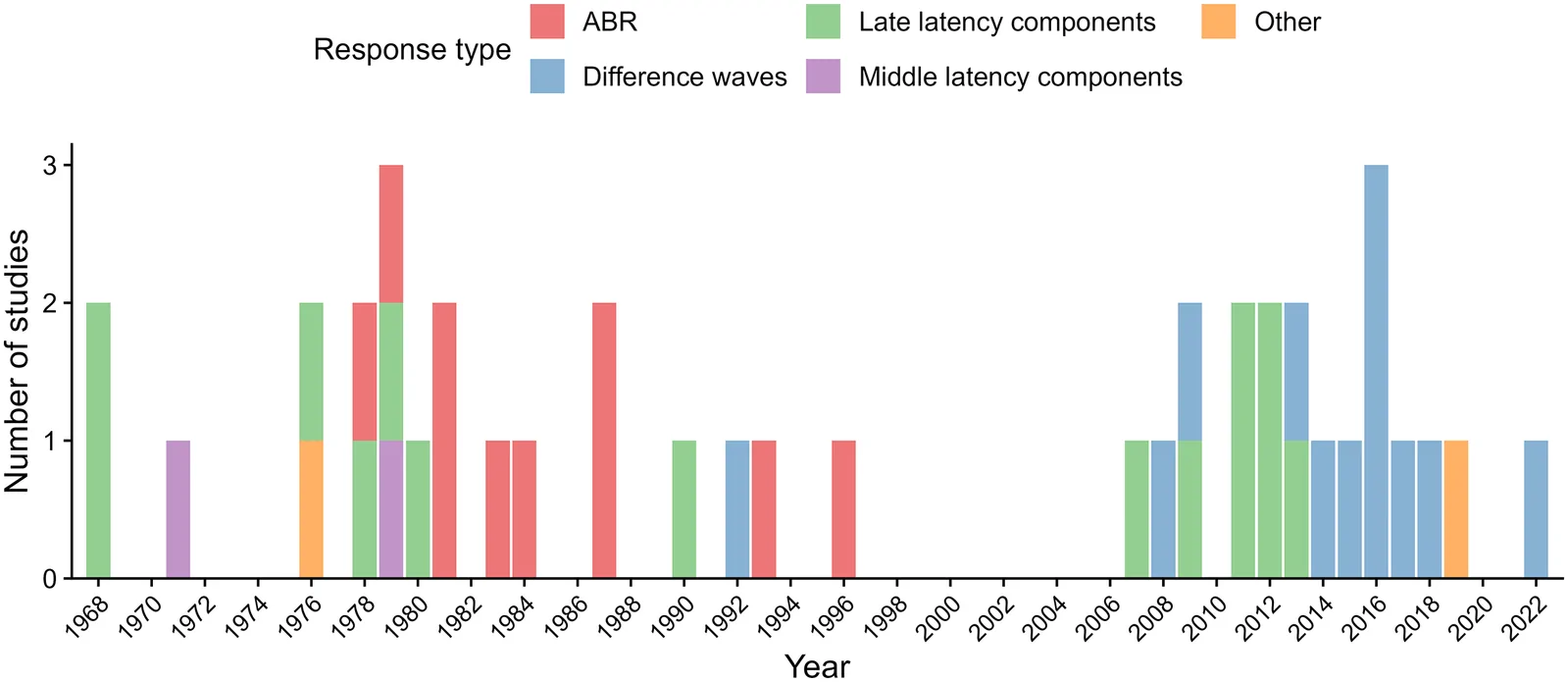
Histogram of RT studies over years. Studies implicated different response types marked by different colors.

The included studies were grouped according to EEG responses studied and organized in S2 Table and in text according to the latency of the brain responses: (1) early latency components (auditory brainstem response, N = 10), (2) middle latency components (Na, Pa, Nb, N = 2), (3) late latency components (P1, N1, P2, N2, P3, N = 14), (4) difference waves studies (mismatch negativity or mismatch response (MMN and MMR), later positive shift, late discriminative negativity (LDN) – 13 studies) and others (one study on Auditory Steady-State Response (ASSR) and one on mean cortical desynchronization). Some studies described more than one category of EEG-response and were therefore reviewed in several sections. Below we describe the results separately for each category.

### Early latency components (auditory brainstem response, ABR)

Auditory brainstem response (ABR) is an early potential that occurs in response to auditory stimuli, reflecting neural activity along the auditory pathways from the inner ear to the brainstem. ABR consists of a series of waves (denoted as I–VII) that appear within the first 10–15 ms after the sound stimulus onset and reflect early auditory processing steps. As ABR occurs within such early latencies, the typical RT studied in this paradigm varies from 0.5 to 5 ms. RT has been studied with ABR for a relatively long time, and no modern studies relevant to the review were found. The most recent study included in this review dates back to 1996. The final review included 10 studies all but one conducted on adults. A single infant study reported no differences from adult findings [13].

One of the main findings consistently reported across studies concerns the latency and amplitude of wave V: as RT increases, wave V latency generally increases, whereas its amplitude decreases [13–17]. This pattern, however, is not universal across all brainstem components and studies. For example, Cobb et al. reported a non-monotonic effect on the latency of waves VI–VII. At 40 dB SPL, increasing RT from 10 µs (“fast rise time”) to 0.5 ms was associated with a decrease in latency, whereas further prolongation of RT up to 5 ms resulted in a gradual increase in latency; however, the latter effect did not reach statistical significance [18]. In the study of Hecox & Deegan (1983), an increase in wave V latency with RT prolongation was also found in most experimental conditions; however, no significant amplitude changes were observed [19]. The study by Suzuki & Horiuchi (1981) was distinguished by its findings of different tendencies depending on the stimuli presented. When utilizing 2–kHz tone pips, a decrease in wave V latency with RT increase was observed at certain stimulus intensities (only 30–40 dB nHL). At the same time, an RT change did not significantly affect wave V amplitude. In contrast, with 0.5–kHz tone pips, an increase in stimulus RT correlated with an increase in the mean wave V amplitude and concurrently, the mean wave V latency also exhibited a tendency to increase. However, these results should be treated with caution as they were obtained on a limited sample (N = 8, all females) in specific conditions: during sleep induced by administration of pentobarbital calcium before the test [20].

One of the articles reported an offset brainstem response that was morphologically similar to the onset brainstem response. This offset response occurred within 8 ms after stimulus offset for tone bursts exceeding 8 ms in duration [21]. Offset ABR was even more affected by stimulus RT than onset ABR. In one experiment, where RT was varied across four values (0.5, 1.0, 2.5, and 5.0 ms), both offset and onset wave V amplitude decreased and latency increased with longer RT. However, due to an increasing number of missing responses at longer RTs, only the 0.5 and 1.0 ms conditions were included in the statistical analysis; the 2.5 and 5.0 ms conditions were excluded [21]. In the other experiment, where RT was varied across two values (0.5 and 5 ms), significant effects were observed only for the offset ABR: offset wave V latency increased for both 500 Hz and 2000 Hz stimuli, and offset amplitude decreased for 2000 Hz stimuli as rise time increased from 0.5 to 5.0 ms [21]. Van Campen et al. proposed that the offset ABR is generated by a more apical region of the cochlea, which would explain its greater sensitivity to RT and its stronger elicitation by low-frequency stimuli.

Stimulus polarity is a physical characteristic that might influence the RT effect. It refers to the initial deflection of the transducer diaphragm in relation to the tympanic membrane during stimulus presentation: a rarefaction stimulus causes the earphone diaphragm to move outward initially, resulting in an outward movement of the tympanic membrane, while a condensation polarity stimulus leads to an inward movement of the diaphragm and consequently an inward movement of the tympanic membrane [22]. Most ABR studies included in the current review used alternating stimulus polarity (rarefaction and condensation), thereby cancelling out the polarity-specific effect. Nonetheless, some investigations within the ABR-field have explicitly considered stimulus polarity as one of the influential acoustic parameters, alongside other stimulus characteristics. Salt & Thornton [23] found that wave I, II, III, and VI amplitudes decreased with RT for rarefaction stimuli but increased for condensation stimuli, though statistical testing was not applied. Gerull et al. [17], however, reported consistent amplitude decreases and latency increases for both polarities, with condensation stimuli producing longer latencies across all RT conditions. Thus, current results on this issue are inconclusive.

In summary, the reviewed ABR studies consistently show that RT prolongation increases the latency and decreases the amplitude of wave V, with similar effects observed for other ABR waves. These findings were robust across stimulus types (tones, noise bursts, clicks) and were observed in both adults and infants, with no age-related differences reported. For an overview of all RT conditions used across the reviewed ABR studies, see Table S2.

### Middle latency components

Middle latency components (10–80 ms post-stimulus) are considered to be driven by the primary auditory cortex with a substantial contribution from thalamocortical pathways, with successive peaks P0, Na, Pa, Nb, Pb [24]. Two studies [16, 25] examined RT effects on these components in neurotypical adults using tone bursts with short RTs (up to 25 ms). Skinner and Antinoro [25] reported that peak-to-peak amplitude of Na–Pa and Pa–Nb components decreased as RT increased across all conditions, while peak-to-peak amplitude of P0–Na and Nb–Pb components decreased only in the longest RT condition (25 ms). Notably, no statistical analyses were used in this study. Kodera et al. (1979) [16] later confirmed statistically the decrease in Na–Pa and Pa–Nb peak-to-peak amplitudes and also showed a delay in the Na and Pa component latencies with RT prolongation. No studies have examined RT effects on middle latency components in children or clinical populations.

### P1

The P1 component is a positive peak in the auditory ERP, occurring approximately 50–100 ms post-stimulus in adults (>100 ms in children), and originating primarily in the primary and secondary auditory cortex. In neurophysiological research, P1 is optimally recorded from central midline electrodes, particularly Cz referenced to mastoids or earlobes, and serves as a biomarker for auditory cortical development and central auditory pathway integrity [26]. We found only three studies that examine P1 modulation by RT which might be related to the fact that P1 is not very prominent in adults.

Stefanics and colleagues [27] examined P1 modulation by RT duration in children with dyslexia and typically reading children (aged 8–10 years) in a longitudinal study that includes oddball and block conditions. A decrease in P1 amplitude with RT increase was observed only in the group of participants with dyslexia, but not in the control group, where P1 amplitudes were equivalent across RT conditions. This effect was specific to oddball conditions; no significant effects were observed for P1 amplitude in the blocked condition. The authors attribute this difference to the greater responsiveness of the P1 component in the oddball condition, which was related to the different probability of occurrence of the stimuli. Both groups demonstrated a prolongation in P1 latency with RT increasing in all conditions. The same prolongation was observed in Kodera et al. (1979b) [28]. In this study P1 was considered as part of the P1–N1 peak-to-peak amplitude, which demonstrates a significant decrease with RT prolongation. In Skinner and Jones’s (1968) [29] work no significant effects for P1 latency were observed. Overall, P1 amplitude effects may be specific to atypical auditory processing (dyslexia), while latency prolongation appears to be a general feature of RT processing across ages.

### N1

The N1 (or N100) is a prominent negative-going component of the auditory ERP waveform that follows P1 and typically peaks approximately 80–120 ms after stimulus onset. It is primarily generated in the auditory cortex, including areas within the superior temporal gyrus (such as Heschl’s gyrus and the planum temporale). In EEG recordings, the N1 component typically exhibits maximal amplitude over fronto-central scalp locations (Fz, FCz, and Cz) [30].

Of all the articles included in the review, thirteen examined the effects of RT on the N1 component. According to the reviewed studies, a reduction in N1 amplitude with RT prolongation is a common pattern for neurotypical adults [28, 31–36], with effects observed across RT differences from 3 ms [32] to 185 ms [31]. In children, Hämäläinen et al. [37] found N1 amplitude decreases in typically developing participants but not in those with reading disabilities. In one of the studies [28], N1 was assessed as P1–N1 peak-to-peak amplitude, which also demonstrates a significant decrease with RT prolongation from 5 to 20 ms.

Three studies reported significant enhancement of N1 latency with RT increase [28, 33, 34]. A similar result was shown in earlier papers [36, 38], but was not confirmed by statistical analysis. In the study by Eswar and colleagues [39], the difference in N1 latency between RT conditions was not significant, which was probably related to minor differences between RT conditions used in this work (7.5 vs 20 ms). However, an even larger contrast (such as 2.5 vs 50 ms) was also insignificant for 1000 Hz tone bursts and white noise bursts [35].

Two studies examined N1 modulation by RT in a group with speech impairment. In Hämäläinen et al. (2007) [37] for a pair of stimuli with a short within-pair interval, the amplitude decreases in the longer RT condition (80 ms), but only in typically developing (TD) children. In the group of children with reading disabilities, the N1 amplitude was equal across RT conditions. It is important to note that stimuli differed not only in RT but also in frequency and statistical comparison of different RT conditions was not performed. The more recent work of this group [33] with stimuli of equal frequencies showed a decrease in N1 amplitude and prolongation of its latency with RT increase in both typical reading and dyslexic adult groups.

In summary, N1 decreases in amplitude and increases in latency with RT prolongation in neurotypical adults. This pattern is less consistent in children and in individuals with dyslexia, where the immaturity or dysfunction of the auditory cortex may modulate these effects.

### P2

The P2 component is a positive deflection in an ERP that follows the N1 component and occurs approximately 150–200 ms post-stimulus. It is generated in the associative auditory cortex and surrounding areas and thus, is typically recorded from central and frontocentral electrode sites (Cz, FCz). This P2 is suggested to reflect higher-order sound processing, auditory feature integration, and early attentional mechanisms [30].

Nine of the papers included in the review consider RT effects associated with the P2 component. Most works estimated P2 effects in terms of N1–P2 peak-to-peak amplitudes [28,34–36,39–41]. N1–P2 amplitude tends to decrease with prolongation of the RT, but only when the difference between RT conditions is sufficiently large. Onishi & Davis [36] observed N1-P2 decrease only when RTs were greater than 30 ms., but no statistical analysis was performed. In Kodera et al. (1979) [28] no significant difference was shown for N1–P2, but RT durations used in this study were quite short (5–20 ms), which was probably not enough for N1–P2 modulation. A similar result appears in a more recent study [39], where no difference was found between 7.5 and 20 ms RT conditions for a 1000 Hz tone burst. Prasher [35] observed significant decreases in P2 for 2.5 ms vs. 50 ms RT for a 1000 Hz tone burst, but not for a white-noise burst. In the study by Elfner et al. [41], a decrease in N1–P2 amplitude was shown for the RT prolongation from 50 ms to 150 ms and 500 ms, but not beyond from 500 to 1500 and 4960 ms, however this finding appeared to be not statistically significant. Two studies [34, 40] used more natural types of stimuli, such as speech tokens and syllables. N1–P2 amplitude decreased with longer RT (such as 79.1 ms in Easwar et al.; in Carpenter & Shanin the exact RT was not reported) in both studies. However, the effect observed in adults and 6-year-old children was not present in early childhood (4–5 years) [40].

Two studies [32, 33] considered P2 as an absolute baseline-to-peak value. Putnam & Roth [32] showed a linear decrease of P2 amplitude (P190 component in the original text) with RT prolongation that included RTs ranging from 3 to 45 ms. This result was confirmed in the study by Hämäläinen and colleagues (2011) [33] for both control and dyslexic adult groups with RTs ranging from 10 to 120 ms.

P2 latency demonstrated a delay with RT prolongation [28, 34]. At first, this effect was described in [38] as the shifting of the slow V potential (N1–P2 peak-to-peak component), but was not statistically validated; later it was confirmed statistically [28, 34]. Nonetheless, in some papers, the differences in P2 latency between different RT conditions were not significant [33,39].

In summary, P2 (or N1–P2 peak-to-peak amplitude), similar to N1, decreases with RT prolongation in adults, with effects typically observed for RT differences exceeding 30 ms. While there is only one study in children, RT effects on N1–P2 amplitude seems to emerge after 6 years of age, reflecting the developmental maturation of auditory processing.

### T-complex

The T-complex captures N1 subcomponents recorded over temporal electrodes (e.g., T7/T8): Na (N1a, 75–95 ms), Ta (N1b, 100–115 ms), and Tb (N1c, 130–170 ms) [42]. This should not be confused with the earlier MLR Na wave (~20–30 ms). The T-complex reflects activity in lateral superior temporal gyrus and planum temporale [42, 43]. Two of the studies included in the review examined T-complex RT. In the study of Hämäläinen et al. (2011) that included neurotypical adults and adults with dyslexia, Na and Ta amplitude did not change between RT conditions in either group, while the amplitude of Tb decreased with RT prolongation only in adults with dyslexia. There was also a prolongation in Na component latency in both groups. In a group of children (7–10 years) Stefanics and colleagues [27] also showed a decrease of N1c (at FT7–FT8 sites) amplitude with long (90 ms) RT in the oddball condition, but in the block condition RT effects were observed only in the dyslexic group. No RT effects on N1c latency were observed.

In summary, the T-complex subcomponents show differential sensitivity to RT: while Na and Ta amplitudes are unaffected by RT in both adults and children, Tb amplitude decreases with RT prolongation in adults with dyslexia and in children, suggesting that Tb may be a marker of atypical or immature auditory processing.

### N2

The N2 component is a negative deflection in the auditory ERP that follows the P2 component and occurs approximately 200–250 ms post-stimulus onset. It originates primarily from frontal cortical regions including the anterior cingulate cortex, which is typically recorded at frontocentral electrode sites (Fz, FCz) [44]. Only one study [27] specifically examined RT effects on N2 in children (7–11 years). Similar to the other components, N2 demonstrated a decrease in amplitude and a prolongation of latency with the RT increase. One more study by Skinner & Jones [29] considered N2 as part of the P1–N2 complex, where the difference between P1 and N2 components was taken as the amplitude of the auditory evoked response. It was shown that P1–N2 decreases with RT increase. N2 latency did not show RT effects, but statistical analyses were not performed. Even though the effects of RT on the N2 component were not considered in other works, we can assume a decrease in amplitude and a latency delay when the RT is prolonged by looking at the figures [33,34].

In summary, the limited evidence suggests that N2 amplitude decreases and N2 latency increases with RT prolongation in children. No studies have examined N2 RT effects in adults.

### P3

The P3 (or P300) component is a prominent positive wave occurring approximately 250–500 ms following auditory stimulus presentation, generated by distributed neural networks involving frontal, temporal and parietal cortical regions. P3 is reliably recorded at midline electrodes (Fz, Cz, Pz) with maximum amplitude typically at Pz [45]. Only Putnam & Roth [32] examined P3 RT effects in adults, finding that P3 amplitude significantly decreased with RT prolongation from 3 to 45 ms. However, this effect may be confounded by simultaneous changes in total stimulus duration. No studies have examined P3 RT effects in children or clinical populations.

### Difference waves (MMN, MMR, late positive shift, LDN)

Many studies investigating RT processing have employed the oddball paradigm, where deviant stimuli with different RTs are embedded within streams of standard stimuli with fixed RTs [31, 46–48]. These deviant stimuli typically elicit a mismatch negativity (MMN) or mismatch response (MMR). MMN is obtained as the difference between the ERP in response to standard (frequent) stimuli and the ERP in response to deviant (rare) stimuli. MMR refers to the corresponding response in infants and young children, which can be positive (pMMR) or negative (nMMR) in polarity, with a broader scalp distribution and more variable latency than adult MMN, reflecting the developing auditory system [49]. Twelve of the reviewed papers examined sensitivity to RT changes using this paradigm, with the MMN component serving as the primary measure of distinguishability between standard and deviant stimuli. Two studies also considered late discriminative negativity (LDN), a later (from 400 ms post-stimulus) component in the difference wave. Although LDN was originally called the late MMN [50, 51], the included studies considered it as a separate component to highlight the difference in the time of occurrence and to separate the RT effects.

The first study [46], conducted in neurotypical adults, demonstrated that RT deviants (24 ms in standards vs. 2 ms in deviants) elicited both an MMN in the 150–250 ms window and a late positive shift in the difference waves in the 280–500 ms window. A later study of neurotypical adults [31] used two RT durations (15 and 185 ms). Each type of stimulus was used as a standard and as a deviant in different blocks. Both types of deviant elicited MMN response (in the time window 150–250 ms) in frontal and temporal areas. In the second part of this study, three types of stimuli were used: one with a long RT (50 ms) and two with an RT of 15 ms (one of them was intensity-matched to the stimulus with 50 ms RT, and the other was not). This contrast was introduced to separate the influences of intensity and RT effects, as changing RT inevitably influences intensity especially with long RT. When 50 ms RT stimuli were used as standard, an intensity-not-matched deviant with shorter RT elicited an MMN response at the time window 175–275 ms (but not significantly), while an intensity-matched deviant did not elicit any MMN at all. However, in the exchanged condition where intensity-matched 15 ms RT stimuli were used as standards and 50 ms RT stimuli were used as deviants, a mismatch response was observed in the time window of 175–275 ms even for the intensity-matched condition. According to these results, the authors suggested that MMN is sensitive to the “content” of the change (RT) but not purely to the amount of stimulus energy (intensity). Meanwhile the context effect – i.e., the observation of a prominent MMN only for conditions with a longer RT for the deviant than the standard – was left unexplained.

Controversial results were obtained concerning the MMN to RT deviant as neuromarker for reading difficulties. The first study [52] considered MMN in response to deviants with short RTs (10 vs 130 ms RT in standard stimuli) in typically reading children and children with reading disabilities (RD) (8–10 years). The study included two conditions with different intervals between stimuli in the presented pair. The authors chose a varying Within-Pair Interval (WPI) design to investigate if children with reading problems exhibit deficits in rapid auditory processing, expecting larger differences with short WPIs, and also to determine if they process rise times differently at an early stage, regardless of the WPI length. This approach allowed them to examine how both rapid presentation rates and RTs variations influence early neural responses in children with and without reading difficulties. Each trial presented a pair of tones under two timing conditions: a short gap condition with 10 ms between tones WPI and a longer gap condition with 255 ms between tones WPI. The experimental design included 80% standard pairs (where both tones had expected properties) and 20% deviant pairs. In deviant pairs, the second tone differed either in RT (10% of trials, 10 ms instead of 130 ms) or in pitch (10% of trials, 750 Hz instead of 500 Hz). The first tone remained consistent across all pairs (100 ms duration, 80 ms rise time, 300 Hz frequency), while the second tone in standard pairs had 150 ms duration, 130 ms RT, and 500 Hz frequency. All tone pairs were separated by a consistent 610 ms interval (ISI), regardless of condition. While in the longer interval condition (255 ms), the MMN response (at latency 119 ms from the deviancy onset) was larger than in the shorter interval (10 ms) in both groups, children with RD demonstrated a larger MMN than typically developing (TD) children only in this longer interval condition. This study also considered LDN. In conditions with a short (10 ms) interval after the previous stimulus, the LDN was not presented. In long within-pair interval (255 ms) conditions, an LDN response was observed at 375–645 ms. In this condition, the control group showed a larger LDN than children with RD.

A traditional non-paired oddball design was employed by Plakas et al. (2013) [47] in their study of younger children (3 years old). A negative mismatch response at 346 ms for longer RT deviants (90 vs 15 ms RT in standard stimuli) was observed in the control group but not in people with dyslexia and typical reading, but with familial risk groups. The study by Hakvoort and colleagues (Hakvoort et al., 2015) involved similar groups of children, but the age of the participants was older (11 years). MMN and LDN, responses peaking around 260 ms and 420 ms respectively to RT deviants with longer RT (90, 180 or 270 vs 15 ms RT in standard stimuli) were observed for all groups (typically developing children, children with dyslexia, and children with familial risk of dyslexia) and did not differ between groups. The influence of different RT durations of deviant stimuli was not considered in this study. The interstimulus interval in this study was close to the long within-pair intervals condition in the Hämäläinen et al. (2008) study (250 ms). These findings suggest that the presence and magnitude of the MMN and LDN component may depend on the timing between stimuli and vary across populations with reading difficulties.

A number of studies have used speech stimuli [3, 53–55]. In these studies, the amplitude RT changes are usually contrasted with the formant RT changes. The syllables /ba/ and /wa/ are commonly used stimuli in this type of study. Syllable /ba/ has a short amplitude RT (10 ms) and formant RT (30 ms), /wa/ has more prolonged amplitude RT (70 ms) and formant RT (110 ms). The third synthetic token usually used in this approach combines a /ba/-like formant RT (30 ms) with a /wa/-like amplitude RT (70 ms). The Moberly et al. study [3] found that while neurotypical adults show MMN response between 150–350 ms for changes in both amplitude and formant RT deviants, the MMN is significantly larger and more consistently observed across individuals for formant RT deviants. In a further study by this research group [53], similar experimental paradigms were used for cochlear implant sample. An MMN response was observed for both deviants, and did not differ between contrasts. Peter et al. [54] implemented similar experimental design for typically developing children and those with dyslexia (mean age = 8.9 years). This study included additional tokens with partial amplitude (40 ms) and formant (70 ms) RT as deviants. Amplitude RT deviants did not elicit any mismatch response in either group. In the control group, both partial and full formant RT deviants produced mismatch effects at a time window of 200–300 ms whereas children with dyslexia exhibited a mismatch response only to the full formant deviants. The developmental track of sensitivity was considered in a follow-up study by this research group [55], including three age groups of children (6–8, 8–10, 10–12 years) and adults. Сhildren under 10 years showed a positive mismatch response between 200 and 400 ms to both types of deviants while 10–12-year-old children and adults were sensitive only to formant RT deviants.

Two studies [56, 57] examined the MMN response to Cantonese syllables with different RTs (/fu/ with RT 120 and 70 ms). In [56], the deviant with 70 ms RT elicited more positive mismatch responses (at latencies about 150–250 ms) than the 120 ms RT deviant. A group of participants with good production and good perception of Cantonese tones showed mismatch responses in both types of counterbalanced contrasts, while a group with good perception but poor production of tones demonstrated mismatch responses only when stimulus with a shorter RT (70 ms) was used as a deviant. In the following paper [57], an additional group with poor speech production and poor speech perception was also included in the analysis. In this group, no significant mismatch responses were observed for either contrast (the 120-ms and 70-ms deviants). The amplitude of the ERP in the time window 50–150 ms (P2) to the stimulus with 70 ms RT was more positive in the good production–good perception group than in the good perception–poor production group and in the poor production–poor perception group. No significant difference between the poor production–good perception group and the poor production–poor perception group was shown. MMN to 120 ms RT deviants did not show any significant difference between groups.

Choisdealbha and colleagues [48] provide a longitudinal study of RT perception involving 7-month infants that was followed four months later. Sine tones and speech-shaped noise were used as stimuli; the RT of standard stimuli was 15 ms and the RT of deviants ranged from 161.1 ms to 292.7 ms, in steps of 14.6 ms. It was shown that larger differences in standard and deviant RT produce a more positive mismatch response (within the time window 300–460 ms). At the same time, the amplitude of the MMR becomes more negative in older infants. Stimulus type did not influence the response. The proportion of infants exhibiting MMN or MMR to deviants with different RTs was broadly similar across all of the used oddball stimuli.

In summary, the presence and magnitude of the MMN/MMR responses both in adults and children depend on the interval between stimuli (ISI) and the specific type of RT manipulated (amplitude vs. formant). In children, the mismatch response undergoes a developmental shift from positive (MMR) to negative (MMN) polarity around 10 years of age. In dyslexia, results are mixed: some studies show reduced or absent mismatch responses, while others show enhanced responses, suggesting that RT processing in dyslexia is complex and may depend on task parameters and developmental stage. Regarding the magnitude of the standard–deviant RT contrast, the values ranged widely across reviewed studies from 22 [55] to 278 ms [51]. Notable, only Choisdealbha et al. [51] systematically varied the RT difference in five steps (161.1–292.7 ms), and demonstrated that larger RT differences elicited larger and more positive mismatch responses.

### ASSR

Van Hirtum et al. examined the auditory steady‑state response (ASSR) – neural activity phase-locked to the modulation frequency of a stimulus – to RT changes in typical readers and adults with dyslexia [58]. They presented amplitude‑modulated white noise at 4, 10, 20, and 40 Hz, with three envelope conditions: a sinusoidal modulation (SAM) as a baseline, and two experimental conditions with RTs of 10 and 30 ms. ASSR magnitude was measured as the signal‑to‑noise ratio (SNR), where nonsynchronized background activity was considered as noise.

Behaviorally, individuals with dyslexia performed similarly to typical readers in discriminating RT and intensity changes, but showed significantly poorer speech‑in‑noise perception. At the neural level, RT‑related group differences were frequency‑specific. Compared to typical readers, participants with dyslexia showed reduced SNRs at 10 Hz for both RT conditions (RT10 and RT30), whereas no group difference was observed for the SAM baseline. At 20 Hz, the SNR reduction was observed only for the longer RT (30 ms), while at 40 Hz it was observed in the right hemisphere for RT10 (RT30 was not tested at this frequency because it was not feasible). No between group differences were found at 4 Hz. In addition to the phase-locked ASSR, neural background activity (i.e., non‑synchronized activity at the corresponding modulation frequency) was also examined. At 40 Hz, right-hemisphere background activity was higher in the dyslexia group and correlated with poorer literacy and phonological skills. In contrast, at 20 Hz, greater beta‑band neural background activity was associated with better literacy skills.

The authors interpreted these frequency‑specific effects in terms of distinct neural mechanisms. The absence of the group differences at 4 Hz is consistent with the role of theta oscillations in tracking syllable‑level rhythm, which may be relatively preserved in dyslexia. In contrast, the reductions of 10 and 20 Hz entrainment were linked to impaired phonological processing. Notably, the finding that the 10 and 20 Hz deficits were modulated by RT suggests that atypical neural synchronization in dyslexia is specifically sensitive to RT cues. The authors conclude that dyslexia is associated with distinct alterations in RT processing at multiple oscillatory frequencies, as well as frequency‑dependent patterns of neural background activity that relate to literacy skills.

### Rhythmic activity

The study by Le Vere and colleagues considered mean cortical desynchronization during sleep in relation to perception of different RTs [59]. Stimuli were random noise centered at 125 Hz with two RT conditions (fast, approximately 3.24 ms, and slow, 7.5 s) and both these fast-rise and slow-rise auditory stimuli could induce arousal during sleep. However, the effectiveness of the stimulus’s RT depended on the sleep stage. During light non-REM sleep (N1–N2, referred to as ‘non-REM fast-wave sleep’ in the original study), both stimulus types were equally effective. In contrast, during slow-wave sleep (N3), a fast-rise stimulus produced significantly more arousal than a slow-rise stimulus, despite the slow-rise stimulus having greater total energy and duration. This highlights the importance of rapid onset, rather than overall energy, in capturing attention and causing arousal during deeper stages of sleep.

## Discussion

In the current review, we present the main studies focused on the electrophysiological correlates of RT perception. While the field under consideration is quite broad and includes both older classical and modern studies, the consistent core finding across most ERP components is a decrease in the amplitude of the main ERP components and an increase in their latencies with RT prolongation. Nevertheless, the observed effects may vary and depend on some aspects of the experimental paradigm, developmental stage and clinical status. Below we discuss these moderating factors, the implications for clinical populations, and directions for future research.

### Stimulation characteristics influencing RT effect

The effect of RT is influenced by other stimulation parameters. Below we discuss some of them and suggest optimal stimulation parameters for experimental design. We conclude this section by presenting differential sensitivity to RT changes at different stages of auditory processing.

### Inter-stimulus interval (ISI)

Different inter-stimulus intervals significantly influence the main ERP components, with component amplitudes generally increasing as the interval lengthens. Moreover, this modulation by stimulus presentation rate varies across developmental stages, reflecting ongoing maturation of neural processing [60]. The reviewed studies used quite variable ISI: from 22 to 457 ms for ABR studies (most often reported as presentation rate); from 500 ms [27] to 14 s [41] for middle and late latency studies; and from 10 ms [52] to 1 s [3, 53] for MMN/MMR studies. However, only two studies compare conditions with different ISI. Hämäläinen et al. [52] examined the effect of the interval between stimuli in a pair, which was equal to 10 or 250 ms (design was described in more detail earlier in the “Difference waves (MMN, MMR, late positive shift, LDN” section in “Results”)). When the within-pair interval was extremely short (10 ms) RT deviants elicited small MMN and did not elicit any LDN, while in the condition with longer 250 ms interval both of these components were observed. Another study by the same group [37] reported larger N1 responses to short (10 ms) compared to long (80 ms) RT in typically developing children in condition with similar short within pair interval (10 ms). However, because the stimuli with different RT also differed in frequency, the observed N1 effect may reflect pitch-related processing rather than RT sensitivity per se – a limitation the authors did not address. At short interstimulus intervals, additional factors such as neural adaptation or overlap between responses to successive stimuli may further obscure rise-time effects.

In summary, we can conclude that an interval of 250 ms is sufficient to detect effects associated with the oddball paradigm (MMN, LDN) [52, 61], and an interval of 500 ms is sufficient to observe effects on middle and late latency components [27, 28], as at shorter intervals the effect may be masked due to stimulus-specific adaptation, while no study assessed it directly.

### Stimulus type

Studies included in the review used different types of stimuli: noise bursts (white noise or speech-shaped noise), sine tones and speech tokens. In the direct comparisons, Prasher’s (1980) [35] found RT effects on N1-P2 amplitude for tones, but not for white noise, attributing this to the absence of spectral transients in noise as tones with short RT (e.g., 2.5 ms) can introduce high-frequency transients due to the abrupt onset, making the stimulus sound sharper or harsher. When longer RTs were used and tone stimuli were contrasted with speech-shaped noise in the sample of infants, no differences between conditions were observed [48]. Other apparent discrepancies – such as the absence of the N1-P2 amplitude modulation for tones, but its presence for syllables – likely reflect methodological differences (overly subtle difference in RT conditions for the tones) rather than inherent limitations of tone stimuli. Indeed, RT effects have been successfully detected with both tones and speech stimuli, and behavioral evidence [62] confirms that RT sensitivity thresholds across stimulus types are significantly correlated, suggesting a common underlying mechanism.

Nonetheless, neural responses to speech sound characteristics suggest that formant RT processing often yields particularly distinct or robust effects in neurotypical populations. In adults, Moberly et al., (2014) found that while MMN responses can be elicited by both amplitude and formant RT deviants, the brain’s response to formant RT changes was stronger and more consistently observed across individuals [3]. This pattern of heightened formant RT sensitivity was absent in adults with cochlear implants [53]. Another study showed that typically developing children exhibited mismatch responses to a broader range of formant RT deviants (including both partial and full changes) compared to children with dyslexia, who demonstrated responses to only full formant RT alterations, thus implying a more finely tuned formant RT processing mechanism in neurotypical peers [54]. Furthermore, developmental research points to an increasing specialization for formant RT in neurotypical individuals with age: children under 10 years respond to both amplitude and formant RT deviants, while older children (i.e., 10–12 years old) and adults show sensitivity primarily to formant RT deviants [55]. Collectively these findings indicate that formant RT serves as a particularly salient acoustic cue for which neurotypical auditory systems develop specialized processing.

### Intensity of stimulation

RT changes inevitably alter cumulative stimulus energy, potentially confounding intensity and RT effects. This effect is associated with the reduction of the plateau duration (the period at which the stimulus has maximal intensity) if RT is increased. Therefore, stimuli with the same duration but different RTs will have different cumulative intensities and can be perceived as stimuli of different loudness. Thomson and colleagues (2009) [31] addressed this directly: when the intensity of the RT deviant was matched across RT conditions, MMN was still observed for intensity-matched deviant but only with a longer than standard RT (50 vs 15 ms), but not in the switched condition (15 vs 50 ms). Thus, MMN is sensitive to the RT per se, but dependent on the context. In an ERP study by Kodera and colleagues (1979) [28] significant RT effects for latency and amplitude of ABR and the main middle and late latency components (except for the N1-P2 peak-to-peak amplitude) were observed only in case of intensity matching. The absence of an effect for the N1-P2 component is quite unusual, as most studies reported these components decrease with RT prolongation. This challenging observation may be related to the fact that when intensity is equated, the main effects of RT are related to the signal detection time. When stimuli are equalized in intensity, the point at which stimuli with different RTs begin to be perceived as different occurs earlier. In this case, such a difference in stimulus detection time could be less critical for later components, especially when the difference between the RT conditions is small (15 ms in Kodera’s work).

RT discrimination thresholds are influenced by the overall stimulus intensity [63]. At low sound levels, the auditory system has less activation, leading to poorer discrimination; as intensity increases, more auditory nerve fibers are recruited, providing a more robust and detailed representation of the sound’s temporal envelope. It is therefore important to consider whether intensity is an essential characteristic for detecting RT effects on the ERP. Some classical auditory perception studies have investigated the effects of RT at different intensities. Onishi and Davis (1968) [36] found that prolongations in N1 latency and the decrease in N1-P2 peak-to-peak amplitude with RT increase were observed at all intensity levels (45, 65, and 85 dB); notably, latency increase was particularly marked at the lowest (45 dB) intensity level. At 15 dB SPL the response was reliably detectable for only one participant in the 3-ms RT condition, and for only two participants in the 30-ms RT condition. Yet N1–P2 amplitude was systematically lower at 3 ms than at 30 ms RTs across all intensities. No interaction between RT and intensity effects was found in the Skinner and Jones study [29]. In Prasher’s study (1980) [35] the observed RT effects did not differ between intensity conditions (80 and 40 dB SPL). However, statistical analyses were limited in early studies.

Overall, no clear association between the observed RT effects and intensity was reported in the studies included in the review. Modulation of amplitude and latency by the RT can be detected even at low intensities (15–45 dB SPL), especially if the RT difference is sufficiently large [25, 29, 36, 41]. Thus, while intensity matching is methodologically important, RT effects are robust to moderate intensity variations. However, there is no effective way to separate the effects of RT and cumulative intensity, as the inevitable matching of cumulative intensity of stimuli with different RT leads to differences in their maximal intensity.

### Differential sensitivity to RT changes across stages of auditory processing

The reviewed studies cover a very wide range of RT from microseconds to hundreds of milliseconds, all of them indexing some aspects of the natural environment. Fast abrupt changes activate the alerting system and are also important for consonant discrimination, RTs between 15–100 ms are crucial for vowel perception, while slower RT changes represent the background auditory scene analysis, emotional component of speech and prosody [64–66].

A summary of differences in RT for different ERPs can be seen in Fig 3C. In most ABR studies RT differences varied from tens of microseconds (the smallest value 90 µs or 0.09 ms in Salt & Thornton study [23]) to 7 ms, while in some studies this difference reached 15 ms. Noteworthy, for each contrast the significant changes in ABR latencies were found, consistent with their role in encoding sound onset in the brainstem pathway. Middle-latency components show sensitivity across 0.5–25 ms RT, reflecting thalamocortical integration over a slightly longer window. The late latency components were sensitive to RT from 5 to 500 ms. While RT differences less than 5 ms (2.49 ms in Prasher’s study, 1980 [35]) and more than 500 ms (in Elfner’s study, 1976 [41]) showed no significant changes in amplitude or latency of these components. The optimal RT ranges also differed within late latency components, being smaller for N1 (>5 ms) and larger for P2 (>30 ms).

**Fig 3:**
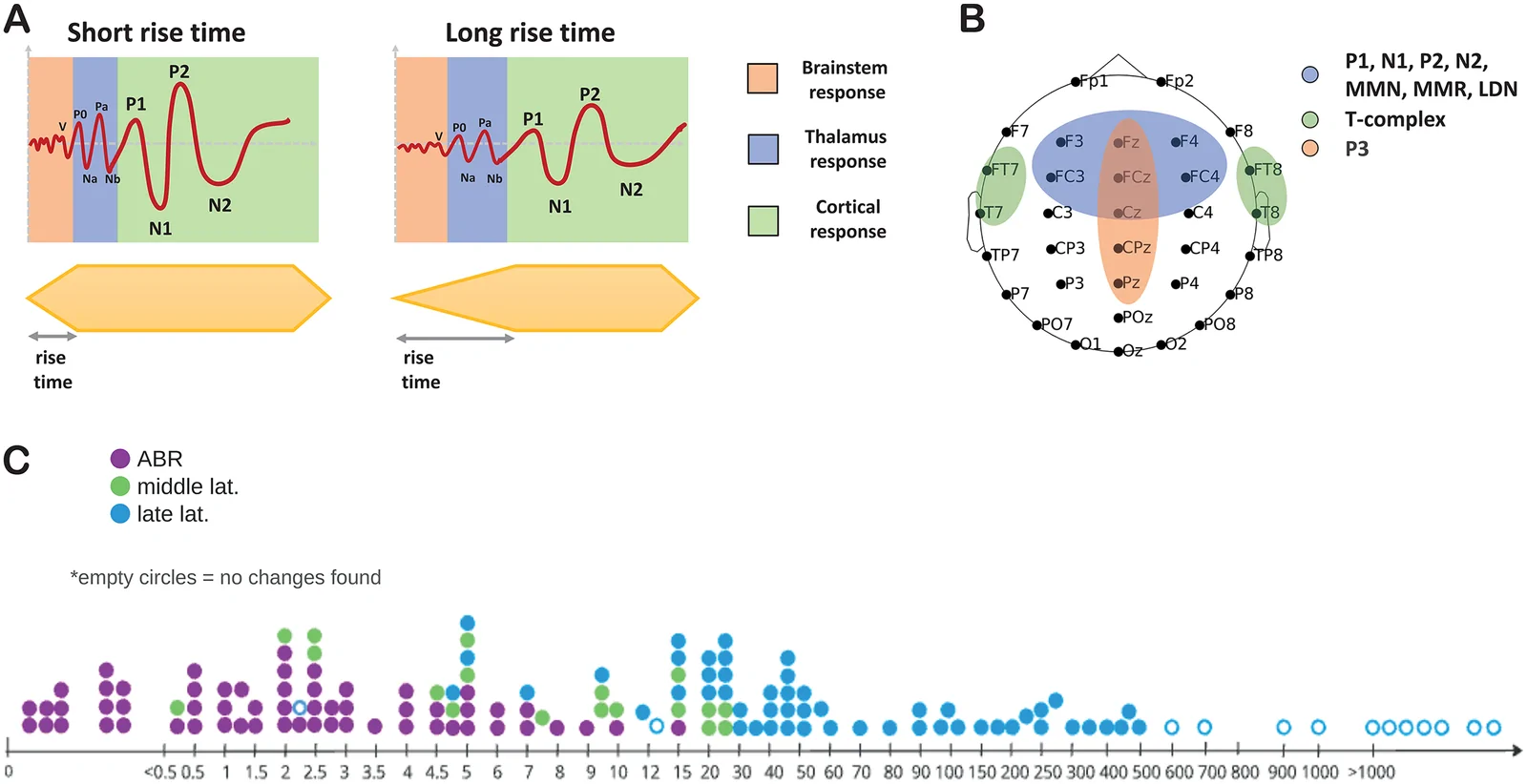
Summary of RT effects on auditory ERP components and typical electrode locations. (A) Schematic illustration of the relationship between RT duration and the amplitude and latency of the main auditory ERP components. Short RTs (left) produce a more abrupt onset, resulting in larger amplitudes and shorter latencies. Long RTs (right) produce a more gradual onset, resulting in smaller amplitudes and prolonged latencies. (B) Typical scalp distribution of the main ERP components discussed in the review. Topographic maps show the approximate electrode locations where each component is optimally recorded. (C) Differences in RT (in ms) in studies observed.

Consequently, to observe significant effects, a more substantial difference in the RT between conditions – typically exceeding 15 ms – is required for the distinctions to be sufficiently pronounced to modulate cortical responses. This extended temporal requirement reflects the cortical areas’ role in integrating information over longer durations for perceptual discrimination, language processing, and cognitive functions, where the precise, rapid onset information critical for brainstem responses has already been processed and is being interpreted within a broader temporal context. A baseline of 15 ms is a standard in cortical RT studies [27, 31, 47, 48, 61]. This value avoids spectral splatter (which occurs with very short RTs) while maintaining temporal precision [67], and aligns with critical RTs in the speech envelope that impact intelligibility [68]. In behavioral tasks, RTs are typically spaced logarithmically from this baseline in staircase procedures [62]. Overall, the observed gradient – from brainstem to cortex – reflects the increasing temporal integration windows at successive levels of auditory hierarchy (Fig 3).

### Developmental changes in electrophysiological markers of rise time perception

Behavioral studies have established that discriminatory sensitivity to acoustic RT changes throughout ontogenesis [62]. In particular, six-year-old children have significantly lower RT thresholds (83.6 ms for sine tones, 150.26 ms for noise, 61.06 ms for speech tokens) than four-year-olds (205.37 ms for sine tones, 257.69 ms for noise, 105.91 ms for speech tokens). However, the neurophysiological underpinnings of this developmental trajectory remain insufficiently characterized, largely due to a scarcity of research focused specifically in children.

The main ERP components undergo significant developmental changes across the lifespan, reflecting the maturation of the auditory processing system. Even the earliest, subcortical auditory responses like ABR demonstrate significant age-related changes, with research showing progressive increases in peak latencies of waves I, III, and V through ontogenesis [69]. A systematic search, however, found only one RT study in the pediatric population that examined ABR: the results for infants in this study were no different from those for adults [13]. No RT studies for children concerning middle latency potentials were found. Late latency components demonstrate prolonged development. In early childhood, auditory evoked potentials are dominated by the P1 and N2 components, while the N1 and P2 components become more prominent with maturation at about 10–12 years of age. Also, the latency of such components decreases with age. While studies of neurotypical adults have demonstrated that the main effects of RT on N1 and P2 components, they cannot be directly generalized to young children as these components are not yet fully developed [70], which suggests that the typical pattern of ERP component modulation by the RT may change with age. Only three studies included in the review examined the RT effects on main ERP components in children [27, 37, 40]. Two of them considered RT effects on N1–P2 peak-to-peak amplitude in children’s samples [37, 40]. In the study by Carpenter & Shanin (2013) [40], RT prolongation leaded to a decrease in N1–P2 amplitude only after 6 years of age, while 4–5-yearold children did not demonstrate any RT effects, which may be related to the poor development of this component in this age group. In the Hämäläinen (2007) [37] study, typically reading children (aged 8.8–10.5 years) demonstrate an N1 decrease with RT prolongation. The third study [27] showed that in children the RT related effects can be transferred to adjacent components (P1 and N2), which probably absorb some functions from not fully developed N1 and P2 components. Indeed, in children (aged 7–11 years), P1 and N2 demonstrates a latency delay and N2 amplitude decrease with RT prolongation

An age-specific phenomenon was also observed for studies with the oddball paradigm. Generally, the latency of the difference-wave mismatch response decreases with age. A decrease in MMN latency in older children is a common fact associated with age-related reduction in the latency of ERP components [71]. This general pattern holds for a deviant stimulus with a different RT. Six of the oddball studies included in this review also examined children. Compared to adult participants, in whom MMN to RT changes were observed in a time window from 150 to 350 ms [3, 46, 53, 56, 57], in children, this time window was shifted [47, 55, 61]. At 11 years the peak of the mismatch response appears at 256 ms [61], while at the age of 3 years – at 346 ms [47]. In infants, the response time window is shifted even more: from 300 ms to 460 ms [48]. Also, age could affect MMN amplitude, which tends to be larger in children than in adults [71, 72]. From the works presented in the review we can also observe the change in the configuration of the mismatch response on the difference wave, which becomes more negative in older participants. The beginnings of these changes can already be observed in infants, but the response remains positive [48]. At around 10 years, the positive MMR is replaced by a negative response, known as MMN [55].

Thus, it is important to take into account that the main target components of ERPs showing RT effects vary quite significantly with age. Thus, the ERP configuration in each age group has to be considered in order to more accurately interpret the RT effects and to be able to compare the results of different studies.

### Rise time perception and speech disorders

Eight of the included studies were focused on the neurophysiological specifics of RT processing in speech disorders (particularly dyslexia). Of these, four examined MMN or MMR using an oddball paradigm [47, 52, 54, 61], three investigated late latency ERP components (such as P1, N1, P2 and N2 [27,33,37]) and one focused on ASSR [58]. The observed results were heterogeneous. Three studies indicated that some patterns of ERP modulation by RT observed in typically-developing participants were absent in individuals with reading disabilities [37, 47, 54]. In contrast, other studies showed that RT-related changes observed in reading disorders were not present in the typically developing group [27, 33, 52]. One study reported no differences between groups at all [61].

Several factors may explain this inconsistency. One of the factors might be the stimulus type. Peter’s (2016) study [54] showed that formant RT MMN might be more informative for dyslexia than amplitude RT neurophysiological response. Developmental stage and genetic predisposition is also crucial. Plakas (2013) [47] showed that three-year-old children with a familial risk of dyslexia, did not exhibit MMR to 90 vs 15 ms RT contracts that were present in peers without such familial risk. This result points towards a genetic predisposition to dyslexia, rather than merely reflecting the diagnosis itself. The absence of group differences by 11 years of age in another study [61] suggests that these early childhood MMR differences might signify a general susceptibility to phonological challenges that could be mitigated by the development of other language skills in older age. Task parameters, such as ISI, are also modulated the results. Hämäläinen and colleagues (2008) [52] indicated a larger MMN to RT changes in children with reading difficulties when preceding stimuli were sufficiently separated. However, this observation was tempered by the suggestion that an elevated N1 component in dyslexia [73] might contribute to this effect through overlap. Collectively, these studies underscore the complex and multifaceted nature of auditory processing deficits in dyslexia, highlighting the need for continued research to refine our understanding and identify robust diagnostic markers.

The reviewed findings suggest potential clinical relevance, but also highlight the current limitations of the evidence. EEG/ERP markers of RT sensitivity, such as reduced N1–P2 amplitude modulation, absent or atypical MMN/MMR responses, have been reported in individuals with dyslexia, but the findings are inconsistent across studies. Such markers could be used for early identification of children at risk for reading impairments or serve as outcome measures for auditory training interventions. For example, reduced MMN/MMR to RT deviants in pre‑reading children has been proposed as a potential early risk marker for dyslexia [47], and prolonged P1 latency or attenuated N1c amplitude in response to RT prolongation may index atypical cortical development [27]. However, the translational value of these findings is currently limited by methodological heterogeneity, small sample sizes, and the lack of standardised protocols. At present, no single EEG/ERP marker of RT processing has been consistently validated across studies, and the available data do not support clinical application. Future research should prioritise replication in large, well‑characterised cohorts and the establishment of normative data before any clinical use can be considered.

### Directions for further research

As previously mentioned, there are certain areas in RT research where the findings are largely consistent, such as a decrease in amplitude and an increase in latency for the main components of ABRs and ERPs. Conversely, the existing results do not permit us to draw clear conclusions about the mechanisms underlying RT effects, particularly in studies related to MMN, LDN, and research involving individuals with dyslexia.

One of the gaps in research revealed by our study is the absence of recent ABR studies (post-1996). Due to methodological limitations of the older studies including outdated technology, small sample sizes, and manual measurements, replication with modern equipment, larger samples, and design-appropriate statistics is warranted. Another clear gap is the absence of research on the main ERP components after 2014, with the field dominated by an oddball paradigm. While the oddball paradigm allows assessing the neurophysiological response to sound differences, it has several drawbacks, such as increased duration of stimulation, a low signal-to-noise ratio, ambiguity in result interpretation, and difficulties in disentangling neural responses to RT changes from responses to other acoustic differences between standard and deviant stimuli [49, 74]. Thus, recent neurophysiological studies on RT can be largely supplemented by the analysis of main ERP components in response to repetitive stimulation.

The third problem is the lack of studies in the pediatric population, especially for basic ERP components, such as P1, N1, P2 and N2, despite their importance for understanding the developmental trajectory of neurophysiological sensitivity to RT, e.g., for linking it with speech perception ability. A similar gap is seen for studies in the clinical groups; only participants with speech and reading difficulties were found to be included in the review.

The reviewed studies have predominantly used isolated syllables or tones to investigate RT processing. However, RT effects in natural, continuous speech can be investigated using recently developed approaches such as temporal response function (TRF) modeling. TRF reconstructs neural responses to continuous acoustic features, including the amplitude envelope and its rise time, and can separate contributions from acoustic and linguistic variables [75]. Extending RT research from isolated stimuli to continuous speech using TRF represents a promising direction, particularly for clinical populations: Power et al. (2016) linked reduced envelope tracking in dyslexia to prosodic impairments [76], and TRF components have been shown to be sensitive to speech-in-noise conditions [77].

As for practical use, the reviewed EEG measures of RT discrimination could be useful for designing more advanced stimulation parameters in auditory brainstem or midbrain implants (ABI/AMI). In cochlear implant users, Moberly et al. (2016) demonstrated that MMN responses to formant and amplitude rise time contrasts can be recorded, and that individuals who weight formant cues similarly to normal‑hearing listeners show temporally segregated MMN responses [53]. This suggests that EEG measures of RT sensitivity are feasible even with degraded peripheral input. For ABI/AMI users, similar approaches could help characterize how the auditory cortex processes implant‑generated signals and whether different stimulation parameters yield more naturalistic cortical response patterns. Nager et al. (2008) recorded MMN in response to RT deviants in a single patient with an auditory midbrain implant, showing that cortical discrimination of RT is preserved even with midbrain stimulation [78]. However, systematic investigations of this kind are lacking, and the use of EEG responses to guide stimulation parameter optimization in ABI/AMI users remains for future research.

Beyond traditional ERP analysis, the reviewed EEG/ERP markers of RT sensitivity may also be integrated into machine learning (ML) frameworks for early screening. For dyslexia, EEG‑based ML models have reported accuracy rates between 82% and 99.2% [79, 80], though most studies rely on resting‑state spectral features rather than auditory ERP measures. At the same time, auditory ERPs have been successfully used to differentiate Rett syndrome from typically developing peers with high accuracy using standard ML models (ROC‑AUC up to 0.94), with P2‑N2 amplitude emerging as a key feature [81]. This suggests that auditory ERP markers can be informative for ML‑based classification, at least in some clinical populations. However, it must be emphasised that, based on the present systematic review, no single EEG/ERP marker of RT processing has been consistently validated across studies. The reviewed evidence reveals considerable heterogeneity in experimental paradigms, stimulus parameters, and populations, and the reported effects vary across components, age groups, and clinical samples. Consequently, the available data do not yet support the use of any specific RT‑related ERP feature as a standalone diagnostic or screening tool. Applying ML approaches to the full set of RT‑related ERP features reviewed here could help identify multivariate patterns that are more robust than any single component, but this remains an open question for future research and requires independent replication in large, well‑characterised cohorts.

### Limitations

Several limitations of this review should be acknowledged. First, our search strategy was restricted to publications in English and to major electronic databases, which may have introduced language and publication biases, potentially omitting relevant studies published elsewhere.

Second, there was substantial methodological and conceptual heterogeneity across studies in some sections. This heterogeneity precluded a quantitative meta-analysis and necessitated a narrative synthesis, which is more susceptible to subjective interpretation. To enhance objectivity, we used a pre-defined framework for synthesis and independent assessment by two reviewers.

Third, the methodological quality of the primary studies was sometimes suboptimal, with frequent unclear or high risk of bias estimates, as assessed by the OHAT tool. Consequently, the overall confidence in the body of evidence for outcomes in some sections, as per our OHAT-based assessment, was not high enough. This inherently qualifies the strength of our conclusions, which should be viewed as hypothesis-generating rather than definitive.

Fourth, a substantial proportion of the reviewed studies had relatively small sample sizes (often N < 15), which may limit the general disability and statistical power of the findings and could contribute to divergent results across studies.

Finally, despite our comprehensive search, the possibility of unpublished null results (publication bias) remains a concern that could skew the presented narrative towards an overestimation of reported effects.

### Declaration of generative AI and AI-assisted technologies in the manuscript preparation process

During the preparation of this manuscript, the authors used GPT 4.0 and DeepL to improve the grammatical accuracy and readability of the English text.

## Supporting information

S1 Table PRISMA checklist. (DOCX)

S2 Table Summary details of the included studies. The studies are divided into groups according to the experimental paradigm used, and organized within each group in ascending order of year.
(DOCX)

S3 Table OHAT risk of bias description and table. (DOCX)
