# Supplementary material for: EEG correlates of auditory rise time processing: A systematic review
Source: PLoS One. 2026 Oct 6;21(10):e0345343. doi: 10.1371/journal.pone.0345343 (PMC13641333; doi:10.1371/journal.pone.0345343)
Supplement: Supplementary file 2 [file pone.0345343.s002.docx]

**Table 1. Summary details of the included studies.** The studies are divided into groups according to the experimental paradigm used, and organized within each group in ascending order of year.

| **Study** | **Sample**  **N (gender)**  **Mean Age (sd)**  **[Age range], years** | **Paradigm** | **Stimuli** | | | **Statistical methods** | **Results (in relation to RT increase)** | **Total risk of bias score** |
| --- | --- | --- | --- | --- | --- | --- | --- | --- |
|  |  |  | **Stimuli type** | **RT** | **Parameters of presentation** |  |  |  |
| Cobb et al. (1978) | N=7 (f:5)  adults | ABR | 1 kHz tones | 0.01, 0.5, 1, 2.5, 5 ms | Binaurally  20, 40, 60 dB SPL  Rate 10/s | Mean, SD | 60 dB:  lat I–VII ↑;  40 dB:  lat I–V ↑; lat VI–VII ↓ between 10 µs and 0.5 ms than ↑ but ns | 6 |
| Stapells & Picton  (1981) | N=11 (f:5)  [22–30] | ABR | **Exp 1–4:** 0.5 kHz tones  **Exp 5:** 0.5, 1, 2, 4 kHz tones  **Exp 6:** +noise  plateau duration: 0.01ms  FT=RT | **Exp 1–4:**  5 ms  **Exp 5–6:**  1, 2, 5, 8 ms | Monaurally (left ear);  115 dB SPL | ANOVA | *amp* V ↓  *lat* V ↑ | 7 |
| Suzuki & Horiuchi (1981) | N=8 (f:8)  [21–34] | ABR | 2 and 0.5 kHz series of tone pips  FT=RT | **2kHZ:**  0.5, 1, 1.5, 2, 3, 5 ms    **0.5 kHz:**  1, 2, 3, 4, 6, 10 ms | Binaurally, earphones  15–50 dB nHL  Rate 13.3/s | Mean, SD | **2kHZ:**  *amp* ns  *lat* V ns on 50 dB nHL  *lat* V ↓ on 30–40 dB nHL  **0.5kHz:**  *amp* V ↑  *lat* V ↑ | 8 |
| Hecox & Deegan (1983) | N=8 (f:8)  **Exp 1:** N=6  **Exp 2:** N=4  [21–27] | ABR | Noise bursts;  Durations 4 and 10 ms  FT=RT | **Exp1:**  0, 1, 2, 5 ms  **Exp2:**  0, 2, 5 ms | Monourally  50 dB above personal threshold (72dB white noise SPL)  Rate 31/s | Pairwise comparison, regression analysis | *amp* ns  *lat* V ↑ | 10 |
| Salt & Thornton (1984) | (N=8) | ABR | Condensation and rarefaction clicks | 0.17–0.58 ms | Monourally (left ear), 106dB SPL  Rate 9.1/s | T-test | *amp* depends on polarity  *lat* I–VI ↑ | 7 |
| Folsom & Aurich (1987) | infants: N=10,  [11–13] weeks,  adults: N=10  [20–30] years | ABR | Gated noise bursts;  plateau duration: 20ms  FT=RT | 0.1, 2.5, 5 ms | Earphones, 50 dB nHL  Rate 16/s | ANOVA | *amp* V ↓  *lat* V ↑ | 10 |
| Gerull et al. (1987) | (N=12)  [25–45] | ABR | Condensation and rarefaction clicks, single-slope stimuli | 0.1, 0.3, 1.0, 3.0 ms | Earphones, 80–120 SPL  Rate 12/s | T-test | *amp* V ↓  *lat* V ↑ | 9 |
| Barth & Burkard (1993) | N=10  adults | ABR | Noise bursts;  Durations 5.0, 5.67, 6.66 and 8.33 s | 0, 0.5, 1.25, 2.5 ms | Monourally  15 dB, 30 dB, 45 dB, 70 dB SPL | Linear regression, rmANOVA | *amp* V ↓  *lat* V ↑ | 10 |
| Van Campen et al. (1996) | N=40 (f:20)  M=26,  [20–30] | ABR | Tone bursts (0.5 and 2 kHz)  Duration 10 ms  FT=RT | 0.5, 1, 2.5, 5 ms | Monourally  103 dB peSPL (peak)  87.5 dB peSPL (peak-to-peak)  Rate 6.3/s | ANOVA | **Exp1 (RT 0.5 VS 1 ms):**  *amp* onset and offset V ↓  (both 0.5 and 2 kHz)  *lat* onset and offset V ↑  **Exp2 (RT 0.5 VS 5 ms):**  *amp* offset V ↓ for 2 kHz  (for 0.5 kHz ns)  *lat* offset V ↑ | 10 |
| Kodera et al. (1979) | N=8  [24-32] | ABR, ERP | 1 kHz tone bursts;  duration 43ms;  FT=RT | 5, 10, 20 ms | Monourally (right ear)  headphones  60 dB SPL for 5ms RT, for other equivalent db SPL  Rate 2/s  order balanced within and between subjects | ANOVA | *amp* ABR↓;  *amp* ABR-Na↓;  *amp* Na-Pa↓;  *amp* Pa-Nb↓;  *amp* P1-N1↓;  *amp* N1-P2: ns  *lat* ABR↑;  *lat* Na↑;  *lat* Pa↑;  *lat* P1↑;  *lat* N1↑  *lat* P2↑ | 7 |
| Skinner & Antinoro (1971) | N=20 (f:12)  [18–24] | ERP | **Exp1:**  1 kHz tone bursts;  plateau duration: 10ms  **Exp2:**  1 kHz tone burst;  durations at 0.01ms RT condition: 0.2, 0.4, 10, 20, 30, 40, and 50 ms;  durations at 5ms RT condition: 1, 5, 10, 20, and 40 ms | **Exp1:**  0.01, 0.5, 2.5, 5, 10, 25 ms  **Exp2**:  0.01 and 5 ms | Monourally  40 dB SPL | NA | **Exp1:**  *amp* Na-Pa and Pa-Nb ↓;  *amp* P0-Na and Nb-Pb ↓ at 25ms RT condition, in other condition ns  **Exp 2:**  *amp* Na-Pa,  Pa-Nb,P0-Na ↓ | 8 |
| Onishi and Davis (1968) | N=7  Adults | ERP | **Exp1:**  1 kHz tone bursts;  plateau durations: 0, 3, 10, 30, 100, and 300 ms;  FT=RT  **Exp2:**  1 kHz tone bursts;  plateau duration: 2.5 ms;  FT=RT | **Exp1:**  3 or 30 ms  **Exp2:**  3, 10, 30, 50, 100, and 300 ms | Binaurally  Earphones,  **Exp1:**  ISI: 2500ms  15–85 dB SPL (varied)  **Exp2:**  ISI: 5000ms  45–85 dB SPL (varied) | only mean and sd | **Exp1:**  *amp* N1-P2 ↓  **Exp2:**  *amp* N1-P2 ↓ (beyond about 30 ms)  *lat* N1 ↑ | 6 |
| Skinner & Jones (1968) | N=40 (f:30)  [18–24] | ERP | 1 kHz tone bursts;  plateau duration: 75 ms | 10 μs, 5, 10, 25, 50 ms | Earphones, 30, 50, 70, and 90 dB SPL  *ISI: 3800ms  random order | only means comparison | *amp* P1-N2↓  *lat* P1 ns  *lat* N2 ns | 8 |
| Elfner et al. (1976) | **Exp1:**  N=7  [18–22]  **Exp2:** N=12  [19–27] | ERP | **Exp1:**  1 kHz sinusoids  durations: 5000 ms;  offset duration: 50 ms  **Exp2:**  1 kHz sinusoids  durations: 1000, 3000, and 5000 ms (random between participants);  offset duration: 50 ms | **Exp1:**  0, 50, 150, 500, 1500, 4960 ms  **Exp2:**  50, 100, 300, 500, 700, 1000 ms | Binaurally  earphones, 45 dB SPL  **Exp1:**  ISI: 10000 ms  **Exp2:**  ISI: 10000, 12000, 14000 ms (random)  RT conditions separate in different trials, trials order randomized | **Exp1:**  NA  **Exp2:** ANOVA | **Exp1:**  *amp* N1-P2↓ from 50 to 500, then ns  **Exp2:**  *amp* N1-P2 ns | 8 |
| Zito (1978) | N=10  adults | ERP | Tone bursts (0.5, 1, 3 kHz);  plateau duration: 200 ms | 5, 250, 500 ms | 70 dB SPL  ISI: 800 ms | NA | *lat* N1↑  *lat* P2↑ | 6 |
| Prasher (1980) | N=15 (f:7)  Adults | ERP | 1 kHz tone and white-noise burst;  duration: 500 ms;  FT=RT | 0.02, 2.5 and 50 ms | Monourally (right ear)  Headphones, 80 and 40 dB SPL  Rate 1/2000 ms  *ISI: 1500 ms | T-test | 2.5ms →50 ms  *amp* N1-P2↓ (for tone, but not for noise);  *lat* N1 ns;  20μs →2.5 ms  *amp* N1P2 ns | 7 |
| Putnam & Roth (1990) | N=16 (f:8)  Mean Age=22  [18–30] | ERP | 1 kHz tones:  durations: 51, 55, 60 and 65 ms (correspond to each RT condition);  FT=RT | 3, 15, 30 and 45 ms | Binaurally  Headphones, 110 dBA SPL  ISI: 8400ms  controlled permutation order  2 runs of paradigm | ANOVA | *amp* N110 ↓ (only 1st run);  *amp* P190 ↓;  *amp* P300 ↓;  Slow wave: ↓; | 10 |
| Hämäläinen et al. (2007) | TD  N=20 (f:7)  Mean Age=9.3(±0.41)  [8.83–10.5]  RD  N=19 (f:10)  Mean Age=9.5 (±0.33)  [8.83–10.5] | ERP  pairs of tones | Tones pairs  1st tone composed of sinusoids (0.3, 0.6, 0.9, and 1.2 kHz)  2nd tone in 5ms RT of sinusoids (0.75, 1.5, 2.25, 3 kHz);  in 80ms RT of sinusoids (0.5, 1, 1.5, and 2 kHz);  1st tone duration: 100ms;  2nd tone duration: 150ms;  FT: 5ms;  WPI: 10 or 130 ms | 1st tone 80 ms;  2nd tone 10 or 80 ms | Binaurally  Loudspeakers, 75 ± 0.5 dB SPL  ISI: 1000-5000 ms | MANOVA | *amp* N1: ↓ TD (In 10ms WPI)  RD ns | 9 |
| Hämäläinen et al. (2011) | TD  N=11 (f:5)  Mean Age=24.5 (±3.9)  Dyslexia  N=11 (f:6)  Mean Age=26.0 (±10.1) | ERP | 0.5 kHz sine tone;  duration: 200 ms;  FT: 50 ms | 10, 30, 60, 90, 120 ms | Binaurally  Headphones, 75 dB SPL  ISI:2500–3500 ms  randomly with equal probability | ANOVA | *amp* N1↓ (both groups);  *amp* P2↓ (both groups);  *amp* Na ns;  *amp* Ta ns;  *amp* Tb↓ (only in  dyslexia);  *lat* N1↑ (both groups);  *lat* P2 ns;  *lat* Na↑ | 11 |
| Stefanics et al. (2011) | TD N=20 (f:13)  Mean Age=8.6 (±0.85) [7.25–10.08]  Dyslexia N=18 (f:9)  Mean Age=9.25 (±1.08) [7.42–11.41] | ERP | 0.5 kHz sine tone;  duration: 200 ms;  FT: 15 ms | standard: 15 ms;  deviant: 90 ms | Binaurally  Headphones, 75 dB SPL  ISI: 500 ms  Oddball condition: (RT, Intensity and Duration deviants)  Blocked condition: (each stimulus type in separate blocks) | ANOVA | **Oddball condition**  *amp* P1 ↓ (in Dyslexia group)  *lat* P1 ↑  *lat* P1 Dyslexia > TD  *amp* N1c (FT7-FT8)↓  *lat* N1c ns  *amp* N2 ↓  *lat* N2 ↑  -MMN  **Blocked condition**  *amp* P1 ns  *lat* P1 ↑  *amp* N1c (FT7-FT8) ↓  *amp* N1c (FT7-FT8) Dyslexia group at 90ms > TD at 15ms  *lat* N1c ns  *amp* N2 ↓  *lat* N2 ns | 12 |
| Easwar et al. (2012a) | N=16 (f: 9)  Mean Age=23.9 [20.3–29.3] | ERP | Syllables (/afil/ and /fi/);  duration: 150 ms | 21.7 and 79.1 ms | Monourally (ears alternated across subjects)  60 dB SPL  Rate 0.49/1000 ms  *ISI: 1853 ms  conditions assessed in separate sessions conducted within a seven-day period | rmANOVA | *amp* N1-P2 ↓  *lat* N1 ↑  *lat* P2 ↑ | 9 |
| Easwar et al., (2012b) | N=16 (f=9)  Mean Age=24.2 (±2.1) | ERP | 1 kHz tone bursts  duration 90 ms  FT=RT | 7.5 and 20 ms | Monourally (ears alternated across subjects)  80 dB SPL  ISI:1910 ms  conditions assessed in separate sessions conducted within a seven-day period | ANOVA | *amp* N1-P2 ns  *lat* N1 ns  *lat* P2 ns | 9 |
| Carpenter and Shahin (2013) | Children  N=15 (f:7)  [4–6.11]  Adult  N=12 (f:6)  [18–25]  for analysis 3 groups:  N=6 [4–5]  N=6 [6]  N=15 [18–25] | ERP | speech tokens  /ba/ and /wa/, and a /ba/ with a /wa/-like RT;  duration 300 ms | NA in text  /ba/ RT< /wa/RT | Binaurally  Earphones, 60–65 dB SPL  ISI: 1500–2000 ms | ANOVA | *amp* N1-P2↓ (in adults and 6 years old, but not in 4-5 year old children) | 11 |
| Lyytinen et al., (1992) | N=6  [18–27] | Oddball,  ERP | 1 kHz tones  duration 50 ms | standard: 24 ms;  deviant: 2 ms | Binaurally  Headphones, 74 dB SPL  ISI: 750 ms  oddball | MANOVA and rmANOVA | +MMN (150-250ms) for RT deviant  +P3, late positive shift (250-450ms) for RT deviant | 10 |
| Hämäläinen et al. (2008) | **Short WPI condition**  TD  N=30 (f:14)  Mean Age=9.4 (±0.25) [8.5–10.0]  RD  N=22 (f:13)  Mean Age=9.4 (±0.33)  [8.8–10.3]  **Long WPI condition**  TD  N=25 (f:13)  Mean Age=9.4 (±0.33)  [8.5–10.0]  RD  N=21 (f:12)  Mean Age=9.4 (±0.33) [8.8–10.3] | Oddball | Pairs of 0.5 kHz tones  1st tone duration: 100 ms;  2nd tone duration: 150 ms;  FT: 5 ms  Short WPI condition: 10 ms;  Long WPI condition: 255 ms | Standard pairs: 1st tone 80 ms, 2nd tone 130 ms  Deviant pairs: 1st tone 80 ms, 2nd tone 10 ms | Binaurally  Loudspeakers, 75 dB SPL  pairs of tones oddball | Temporal principal components analysis (PCA); MANOVA | **Short WPI condition:**  +MMN (124 ms) for RT deviant;  -LDN for RT deviant;  *amp* MMN ↑ in right mastoid (both TD and RD);  amp LDN ns;  **Long WPI condition**  +MMN (119 ms) for RT deviant;  +LDN (375–645 ms) for RT deviant;  MMN RD> TD at fronto-central channels;  LDN TD > RD at right frontal and central  channels and left mastoid channel | 10 |
| Thomson et al. (2009) | **Exp1**  N=12 (f:7)  Mean Age=26  [20–42]  **Exp2**  N=14 (f:11)  Mean Age=26  [20–42] | Oddball | 0.5 kHz sine tones;  duration: 260 ms;  FT: 50 ms  **Exp2**  Standart: 15 or 50 ms IM | **Exp1**  15 and 185 ms  **Exp2**  50 ms, 15 ms intensity matched to 50 ms (IM) and 15 ms intensity not matched (INM) | Binaurally  Headphones, 75 dB SPL  ISI: 240 ms  **Exp1** counterbalanced oddball  **Exp2**  oddball | ANOVA | **Exp1**  amp N1 ↓;  +MMN (150–250 ms) for both deviants;  **Exp2**  amp N1 ↓;  when 50 ms standart:  -MMN for 15 ms IM deviant  +MMN (but ns) (175–275ms) for 15ms INM deviant  when 15ms IM standart  +MMN (175–275ms) for 50ms deviant | 11 |
| Plakas, et al. (2013) | Control  N=13  Typical readers at risk  N=15  Dyslexic at risk  N=10  Mean Age=3,4 | Oddball | 0.5 kHz tone;  duration: 155 ms;  FT: 50 ms | standart 15 ms;  deviant 90 ms | Binaurally  Speakers, 75 dB SPL  ISI: 450 ms  oddball | ANOVA | Control  +MMR (peak at 346 ms) for RT deviant  Typical readers at risk:  –MMR for RT deviant  Dyslexics at risk  –MMR for RT deviant  MMR in control >  MMR at risk  MMR Typical readers at risk =  MMR Dyslexic at risk | 11 |
| Moberly et al., (2014) | N=13 (f=8)  Mean Age=25.2  [19–42] | Oddball | speech tokens  /ba/ and /wa/  ART/ba/–FRT/ba/;  ART/wa/–FRT/wa/;  ART/wa/–FRT/ba/;  duration: 370ms | ART/ba/ 10 ms, ART/wa/70 ms;  FRT/ba/ 30 ms, FRT/wa/110 ms | Binaurally  Free-field, 70 dB SPL  ISI:1000 ms  counterbalanced oddball | ANOVA | +MMN (150–350 ms) for ART deviants  +MMN (150–350 ms) for FRT deviants  MMN for FRT deviant>MMN for ART deviant | 10 |
| Hakvoort, et al. (2015) | Group no FR no dyslexia  N = 15  Mean Age=11.96 (±0.6)  FR no dyslexia  N = 24  Mean Age=11.9 (±0.6)  FR dyslexia N = 15  Mean Age=11.84 (±0.5) | Oddball | 523 Hz sine tone  duration: 400 ms;  FT: 50 ms | standart: 15 ms;  deviant: 90, 180 and 270 ms | 80 dB SPL  ISI: 250 ms  oddball | ANOVA | +MMN (peak at 260 ms) (all groups)  +LDN (peak at 420 ms) (all groups)  no difference between groups | 12 |
| Moberly et al., (2016) | N=20, cochlear implants  [18–62] | Oddball | speech tokens  /ba/ and /wa/;  ART/ba/–FRT/ba/;  ART/wa/–FRT/wa/;  ART/wa/–FRT/ba/;  duration: 370 ms | ART/ba/ 10 ms, ART/wa/ 70 ms;  FRT/ba/ 30 ms, FRT/wa/ 110 ms | Binaurally  Speakers, 68 dB SPL  ISI:1000 ms  counterbalanced oddball | ANOVA | +MMN (150–350 ms) for ART deviants  +MMN (150–350 ms) for FRT deviants  MMN (150–350 ms) for FRT deviant=MMN (150–350 ms) for ART deviant | 11 |
| Ou and Law (2016) | [+Per+Pro]  (Good tone perception and production)  N=20  Mean Age=22.0(±0.59)  [+Per–Pro]  (Good tone perception and poor production)  N=19  Mean Age=21.24 (±0.84) | Oddball | Cantonese tones /fu/;  duration 500 ms | 120 and 70 ms | Binaurally  Earphones, 85 dB SPL  ISI: 800 ms  counterbalanced oddball | ANOVA; cluster-based permutation tests;  T-tests | [+Per+Pro]  +MMN (150–200 ms) when 70 ms deviant  +MMN (150–238 ms) when 120 ms deviant  [+Per–Pro]  +MMN (150–200 ms) when 70 ms deviant  –MMN when 120 ms deviant | 10 |
| Peter. et al., (2016) | Control:  N=17 (f:5)  Dyslexia  N=17 (f:3)  Mean Age=8.9 (±1.4)  [6.0–11.8] | Oddball | speech tokens  /ba/ and /wa/  standart: ART/ba/–FRT/ba/  ART deviant:  ART/midway/–FRT/ba/  (partial deviant)  ART/wa/–FRT/ba/  (full deviant)  FRT deviant:  ART/ba/–FRT/wa/  (full deviant)  ART/ba/–FRT/midway/ (partial deviant)l  duration: 320 ms | ART/ba/ 10 ms,  ART/midway/ 40 ms,  ART/wa/ 70 ms;  FRT/ba/ 30 ms,  FRT/midway/ 70 ms FRT/wa/ 110 ms | Binaurally  Loudspeakers, 75 dB SPL  ISI: 180 ms  oddball | Independent T-test, cluster-based permutation tests | Control:  –MMR for both ART deviants  +MMR (200–300 ms) for both FRT deviants  Dyslexia  –MMR for both ART deviants  –MMR for partial FRT deviant  +MMR (200–300 ms) for full FRT deviant | 12 |
| Ou and Law (2017) | [+Per+Pro]  (Good tone perception and production)  N=20  Mean Age=22.00 (±0.59)  [+Per–Pro]  (Good tone perception and poor production) N=19  Mean Age=21.24 (±0.84)  [–Per–Pro]  (Poor tone perception and production)  N=19  Mean Age=21.20 (±2.84) | Oddball | Cantonese tones /fu/  duration 500 ms | 120 and 70 ms | Binaurally  Earphones, 85 dB SPL  ISI: 800 ms  counterbalanced oddball | ANOVA; cluster-based permutation tests | [+Per+Pro]  +MMN (150–200 ms) when 70 ms deviant  +MMN (150–238 ms) when 120 ms deviant  [+Per–Pro]  +MMN (150–200 ms) when 70 ms deviant  –MMN when 120 ms deviant  [–Per–Pro]  –MMN for both deviants  *amp* P2 ↓ (70 ms generate more positive response then 120 ms)  for 70 ms: [+Per+Pro]>  [+Per–Pro]=[–Per–Pro]  for 120 ms: no difference between groups | 12 |
| Peter, et al. (2018) | 6–8 years  N=12 (f:6)  Mean Age=7.18 (±0.29)  8–10 years  N=12 (f:7)  Mean Age=8.87 (±0.44)  10–12 years  N=12 (f:7)  Mean Age=10.92 (±0.54)  Adults  N=12 (f:6)  Mean Age=31.05 (±4.41) | Oddball | speech tokens  /ba/ and /wa/  standart: ART/ba/–FRT/ba/  ART deviant:  ART/wa/–FRT/ba/  FRT deviant:  ART/ba/–FRT/wa/  duration: 320ms | ART/ba/ 10 ms, ART/wa/ 70 ms;  FRT/ba/ 30 ms, FRT/wa/ 110 ms | Binaurally  Speakers, 75 dB SPL  ISI: 180 ms  oddball | ANOVA; cluster-based permutation tests | 6–8 years  +MMR (200–400 ms) for ART deviant  +MMR (200–400 ms) for FRT deviant  8–10 years  +MMR (200–400 ms) for ART deviant  +MMR (200–400 ms) for FRT deviant  10–12 years  –MMR for ART deviant  +MMR (204–316 ms) for FRT deviant  Adults  –MMR for ART deviant  +MMR (168–248 ms) for FRT deviant | 12 |
| Choisdealbha et al. (2022) | Seven months N=74  Mean Age=7 months, 3 days (±5 days)  Eleven months N=96  Mean Age=11 months, 2 days (±5 days) | Oddball | sine tone or SSN (speech shaped noise) | standart:15 ms  10 deviants: 161.1 –292.7 ms (step of 14.6 ms) | Binaurally  Speakers  oddball | regression model | +MMR (300–460 ms)  MMR↑ with deviant RT↑  MMR more negative with Age↑  MMR for tones=MMN for SSN | 11 |
| Le Vere et al. (1976) | M=20.3  [19–23] | Rhythmic activity | Tones (125 Hz) | 3.24 ms (fast RT) and 7.5s (slow RT) | Auditory speaker system;  free field | ANOVA | At slow wave Mean cortical desynchronization ↓  At fast wave Mean cortical desynchronization nc | 8 |
| Van Hirtum et al. (2019) | Dyslexia:  N=20 (f:10)  [18–25]  Typical readers: N=18 (f:10)  [18–25] | ASSR | amplitude-modulated one-octave white noise bands centered 1 kHz. Noise bands were 100% amplitude-modulated | amplitude-modulated at approximately 4, 10, 20 and 40 Hz (sinusoidal envelope modulation, 10 and 30 ms RT) | Monourally (right ear)  Earphones, 85 dB SPL | linear mixed models, t-test | 4 Hz: ↑ for RT30 and ↓ for RT10  10 Hz: RT10 and RT30 ↓ dyslexia only  20 Hz: RT30 ↓ а only  20 Hz: higher synchronization of beta activity for RT30 related to better literacy skills  40 Hz: ↓ SNR dyslexia only in the right hemisphere  40 Hz: correlation of neural background activity in the right hemisphere with literacy and phonology in participants with dyslexia | 12 |

**List of abbreviations for Table 1**

ABR – auditory brainstem response

ASSR – auditory steady-state response

ERP – event-related potentials

MMN (xxx ms) – mismatch negativity (latency of the component)

MMR (xxx ms) – mismatch response (latency of the component)

LDN (xxx ms) – late discriminative negativity (latency of the component)

RT – rise time

FT – fall time

ART – amplitude rise time

FRT – formant rise time

ISI – inter-stimulus interval

WPI – within-pair interval

lat – latency

amp – amplitude

ns – not significant

I, II, III, IV, V, VI, VII, VIII – number of ABR waves

SPL – sound pressure level

nHL– normalized Hearing Level

TD – typically developing

RD – reading disabilities

FR – family risk (of dyslexia)

+ – the presence of the response

– – the absence of the response

↑ – response increases with increasing of RT increase

↓ – response decreases with increasing of RT increase

Converted values are marked with an asterisk
