# Supplementary material for: EEG correlates of auditory rise time processing: A systematic review
Source: PLoS One. 2026 Oct 6;21(10):e0345343. doi: 10.1371/journal.pone.0345343 (PMC13641333; doi:10.1371/journal.pone.0345343)
Supplement: Supplementary file 3 [file pone.0345343.s003.docx]

The risk of bias of the included studies was assessed according to the OHAT risk of bias tool using the following scale of marks for each question: “++” – definitely low risk of bias; “+” – probably low risk of bias; “–” – probably high risk of bias, “– –” – definitely high risk of bias. For the total score calculation, the OHAT tool marks were translated to values in the following way: “++” – 3 points, “+” – 2 points, “–” – 1 point, “– –” – 0 points. Risk of bias evaluation included 3 sections with several questions inside them: the selection bias, the detection bias, and the “other sources of bias” sections. The attrition/exclusion, selective reporting bias sections and corresponding questions were excluded because these categories are irrelevant for the studies included. The questions of the selection bias section were merged into one assessing adequacy of the randomization and group. Studies were marked with “++” in case of proper randomization of participants groups in terms of general characteristics, such as age, gender, etc.; with “+” in case of mild faults and with “–” in case of not defined age, gender or not enough sample size. The detection bias section consisted of two questions. We modified the question “Can we be confident in the exposure characterization?” to “Can we be confident in the reliability of the rise time condition?”. Studies clearly controlling physical parameters of acoustic stimuli (frequency, intensity, fall times etc.) were marked with “++”; studies controlling not all of the relevant parameters were marked with “+”; studies not controlling relevant parameters at all were marked with “–”. Because we considered only EEG-studies, we modified the question “Can we be confident in the outcome assessment?” to “Can we be confident in EEG parameters assessment?” in order to evaluate the reliability of the tools used to assess neurophysiological parameters. Studies using reliable psychophysiological measurements with enough numbers of trials and сorrect approach to EEG parameters estimation were marked with “++”; studies using less reliable methods were marked with “+”; studies using only one unreliable measure with not enough number of trials were marked with “– –”.

The section “Other sources of bias” was focused on the appropriateness of the statistical methods used. Studies using reliable statistical methods with no visible faults were marked with “++”, with mild inaccuracy – with “+”; studies with an obvious fault (e.g. proceeding to post-hoc analysis after detection a statistical tendency) were marked with “–”; studies with pronounced inaccuracies or absence on statistical analysis were marked with “– –”.

| **Study** | **Selection bias** | **Detection bias** | | **Other sources of bias** | **Total score** |
| --- | --- | --- | --- | --- | --- |
|  | **Can we be confident in randomisation/ group allocation or whether the comparison groups were appropriate?** | **Can we be confident in the reliability of the rise time condition?** | **Can we be confident in EEG parameters assessment?** | **Were the statistical methods used appropriate?** |  |
| [Barth & Burkard (1993)](https://www.zotero.org/google-docs/?Kc01ph) | "-" | "++" | "++" | "++" | 10 |
| Carpenter and Shahin (2013) | "+" | "++" | "++" | "++" | 11 |
| Choisdealbha et al. (2022) | "++" | "+" | "++" | "++" | 11 |
| [Cobb et al. (1978)](https://www.zotero.org/google-docs/?AVb1Ib) | "+" | "++" | "+" | "--" | 7 |
| [Easwar et al. (2012)](https://www.zotero.org/google-docs/?broken=SVFWAb) | "+" | "+" | "+" | "++" | 9 |
| [Easwar et al. (2012)](https://www.zotero.org/google-docs/?broken=SCibac) | "+" | "+" | "+" | "++" | 9 |
| [Elfner et al. (1976)](https://www.zotero.org/google-docs/?broken=2R3Pdm) | "-" | "++" | "+" | "+" | 8 |
| [Folsom & Aurich (1987)](https://www.zotero.org/google-docs/?broken=T3lisZ) | "+" | "++" | "+" | "+" | 10 |
| [Gerull et al. (1987)](https://www.zotero.org/google-docs/?broken=y6cUQo) | "+" | "++" | "+" | "+" | 9 |
| [Hakvoort et al. (2015](https://www.zotero.org/google-docs/?cmFeFr)) | "++" | "++" | "++" | "++" | 12 |
| [Hämäläinen et al. (2008)](https://www.zotero.org/google-docs/?broken=SmliAv) | "++" | "+" | "++" | "+" | 10 |
| [Hämäläinen et al. (2007)](https://www.zotero.org/google-docs/?broken=1ROcNn) | "++" | "-" | "++" | "+" | 9 |
| [Hämäläinen et al. (2011)](https://www.zotero.org/google-docs/?broken=VWjkY7) | "+" | "++" | "++" | "++" | 11 |
| [Hecox & Deegan (1983)](https://www.zotero.org/google-docs/?broken=ryP0hg) | "+" | "+" | "++" | "++" | 10 |
| [Kodera et al. (1979)](https://www.zotero.org/google-docs/?broken=AFEJOr) | "-" | "+" | "+" | "+" | 7 |
| [Le Vere (1976)](https://www.zotero.org/google-docs/?broken=GyTZU5) | "-" | "+" | "++" | "+" | 8 |
| [Lyytinen et al. (1992)](https://www.zotero.org/google-docs/?broken=VRajlM) | "+" | "++" | "+" | "++" | 10 |
| [Moberly et al. (2014)](https://www.zotero.org/google-docs/?broken=Ukrw0c) | "++" | "+" | "++" | "+" | 10 |
| [Moberly et al. (2016)](https://www.zotero.org/google-docs/?broken=i5HDpY) | "++" | "+" | "++" | "++" | 11 |
| [Onishi & Davis (1968)](https://www.zotero.org/google-docs/?broken=CGRFjY) | "-" | "++" | "+" | "--" | 6 |
| [Ou & Law (2017)](https://www.zotero.org/google-docs/?broken=VOGXt1) | "++" | "++" | "++" | "++" | 12 |
| [Ou & Law (2016)](https://www.zotero.org/google-docs/?broken=ny9Ocy) | "-" | "++" | "++" | "++" | 10 |
| [Peter et al. (2016)](https://www.zotero.org/google-docs/?broken=7Wdn3G) | "++" | "++" | "++" | "++" | 12 |
| [Peter et al. (2018)](https://www.zotero.org/google-docs/?broken=EdZr3O) | "++" | "++" | "++" | "++" | 12 |
| [Plakas et al. (2013)](https://www.zotero.org/google-docs/?broken=juWKm3) | "++" | "++" | "+" | "++" | 11 |
| [Prasher (1980)](https://www.zotero.org/google-docs/?broken=3NFEjI) | "-" | "+" | "+" | "+" | 7 |
| [Putnam & Roth (1990)](https://www.zotero.org/google-docs/?broken=qL9ABV) | "+" | "+" | "++" | "++" | 10 |
| [Salt & Thornton (1984)](https://www.zotero.org/google-docs/?broken=3Hqq0D) | "-" | "+" | "+" | "+" | 7 |
| [Skinner & Antinoro (1971)](https://www.zotero.org/google-docs/?broken=MigNet) | "++" | "+" | "++" | "--" | 8 |
| [Skinner & Jones (1968)](https://www.zotero.org/google-docs/?broken=nQ0Jkw) | "++" | "++" | "+" | "--” | 8 |
| [Stapells & Picton (1981)](https://www.zotero.org/google-docs/?broken=s51EAf) | "+" | "+" | "+" | "-" | 7 |
| [Stefanics et al. (2011)](https://www.zotero.org/google-docs/?broken=hxAZJO) | "++" | "++" | "++" | "++" | 12 |
| [Suzuki & Horiuchi (1981)](https://www.zotero.org/google-docs/?broken=0KdT85) | "+" | "+" | "++" | "-" | 8 |
| [Thomson et al. (2009)](https://www.zotero.org/google-docs/?broken=8hAYPv) | "+" | "++" | "++" | "++" | 11 |
| [Van Hirtum et al. (2019)](https://www.zotero.org/google-docs/?broken=8FDxvj) | "++" | "++" | "++" | "++" | 12 |
| [Van Campen et al. (1997)](https://www.zotero.org/google-docs/?broken=SSvm4J) | "++" | "+" | "+" | "++" | 10 |
| [Zito (1978)](https://www.zotero.org/google-docs/?broken=K3AFBL) | "-" | "++" | "+" | "--" | 6 |
