# Supplementary material for: EEG correlates of auditory rise time processing: A systematic review
Source: PLoS One. 2026 Oct 6;21(10):e0345343. doi: 10.1371/journal.pone.0345343 (PMC13641333; doi:10.1371/journal.pone.0345343)
Supplement: Supplementary file 4 [file pone.0345343.s004.docx]

Reviewer Comments Manuscript Number: PONE-D-26-09613 Title: EEG correlates of auditory rise time processing: A systematic review Corresponding Author: Victoria Manasevich

Reviewer: D. Bradley Welling, MD, PhD

Scope of This Review: I was asked to review this manuscript to offer a clinical perspective on its relevance to patient populations within my area of practice. I want to note at the outset that my expertise is clinical rather than in EEG signal-processing methodology, event-related potential paradigm design, or psychophysical experimental methods — which make up the majority of this manuscript's content. My comments below are accordingly limited to (1) clinical relevance and accessibility for a clinical readership, and (2) points of language clarity that affected my ability, as a non-specialist reader, to follow specific sections. I would defer questions of methodological rigor, statistical approach, and completeness of the systematic review process to a reviewer with more direct expertise in EEG/ERP methodology.

General Comments

● Clinical relevance and accessibility. Although the review touches on populations of clinical interest (dyslexia, ASD, speech-language disorders), the manuscript remains oriented primarily toward basic psychophysics and EEG methodology. A clinical reader would benefit from a more explicit paragraph connecting rise-time processing markers to diagnostic or therapeutic relevance.

● Electrode placement schematic. I would suggest adding a figure illustrating typical electrode placement/montage for the ERP components discussed (ABR, MMN, N1P2, etc.). This would help readers less familiar with EEG methodology follow the anatomical and temporal correspondence of the components being compared.

● Abbreviation definitions. Given the density of abbreviations (ABR, ASSR, ERP, MMN, MMR, LDN, RT, FT, and clinical terms such as ASD), I recommend a full pass to confirm every abbreviation is defined at first use in the body text, not only in the supplementary abbreviation table (see line 924 below).

● Length and structure. The manuscript is quite long. The authors may wish to consider whether some of the more granular study-by-study parameter comparisons (e.g., the results subsections cataloguing RTdifference ranges across individual studies) could be moved to supplementary tables, which might improve readability without loss of content.

Specific Comments

Line 234–235 The sentence is difficult to parse: “One of the articles reported morphologically similar to onset brainstem response offset brainstem response which occurs by long-duration tone burst (>8 ms) within 8 ms after stimulus offset [22].”

The sentence needs restructuring. Suggested revision: “One of the articles reported an offset brainstem response that was morphologically similar to the onset brainstem response and occurred within 8 ms of stimulus offset for long-duration tone bursts (>8 ms) [22].”

Lines 695–697 “…the auditory system has fewer activation, leading to poorer discrimination.” ‘Fewer’ does not grammatically modify the uncountable noun ‘activation.’ Suggest ‘less activation’ or, to align with the wording in the following sentence, ‘fewer activated auditory nerve fibers.’

Line 707 “…an intensity condition of 15 was used, at this condition response was above noise level for only one participant…” This is a comma splice; the sentence should be split into two after ‘used.’ Separately, please clarify the units for ‘intensity condition of 15’ (presumably dB SPL or dB HL) — as written it is ambiguous.

Lines 737–739 Subject–verb agreement: “…the components modulation were demonstrated for all used contrasts. The range of RT differences, which showed significant results for late latency (components was from 4.99 ms to 500 ms…” ‘Components was’ should read ‘components were.’

Lines 752–754 “…researchers typically present target sounds gradually logarithmically ranging from 15 ms (the standard) to longer durations.” This phrase is unclear to me as a non-specialist reader. Please clarify whether this refers to stimulus values spaced logarithmically across a study/staircase procedure, or to some other aspect of stimulus presentation.

Line 924 “…with ASD [81].” The abbreviation ‘ASD’ is used here and repeated at lines 926, 928, and 929, but is never spelled out in the body text — it appears written out only within reference titles in the bibliography. Recommend defining ‘Autism Spectrum Disorder (ASD)’ at first use.

Comment

Would any of the parameters summarized in this review be of use with auditory cortical stimulation with AI algorithms in the future? Auditory brainstem implant (ABI) users experience a wide range of open-set speech understanding, with many users unable to benefit substantially. Could future studies utilize the EEG response to ABI signals to better understand the way the auditory cortex is processing these signals and be useful in designing more advanced stimulation parameters similar to the information gleaned from Moberly et al.

I found the information on differences in EEG response in dyslexia interesting and would welcome more information on related topics. The brief reference to Autism Spectrum Disorders should be described in more detail or omitted.

Recommendation: Recognizing that my review is limited to language clarity and clinical-relevance considerations rather than methodological or statistical evaluation, I recommend minor revision, contingent on: (1) a language-editing pass addressing the issues noted above (and likely others outside my ability to catch, given the technical density of the material); (2) clarification of the ambiguous methodological language flagged above; (3) consideration of an electrode-placement schematic; and (4) confirmation from a reviewer with direct EEG/ERP methodological expertise regarding the completeness and rigor of the systematic review process itself, which falls outside my area of qualification to assess.

D. Bradley Welling, MD, PhD
