# Supplementary material for: EEG correlates of auditory rise time processing: A systematic review
Source: PLoS One. 2026 Oct 6;21(10):e0345343. doi: 10.1371/journal.pone.0345343 (PMC13641333; doi:10.1371/journal.pone.0345343)
Supplement: Peer review history

# Peer review: Decision Letter 0

**DOI:** 10.1371/journal.pone.0345343.r001

**Authors:** Stephen Ginsberg

22 Jul 2026

PLOS One

Dear Dr. Manasevich,

Thank you for submitting your manuscript to PLOS ONE. After careful consideration by two Reviewers and an Academic Editor, we feel that it has merit but does not fully meet PLOS ONE’s publication criteria as it currently stands. Therefore, we invite you to submit a revised version of the manuscript that addresses the points raised during the review process.

Please submit your revised manuscript by Sep 05 2026 11:59PM. If you will need more time than this to complete your revisions, please reply to this message or contact the journal office at plosone@plos.org. When you're ready to submit your revision, log on to https://www.editorialmanager.com/pone/ and select the 'Submissions Needing Revision' folder to locate your manuscript file.

Please include the following items when submitting your revised manuscript:

- A letter that responds to each point raised by the academic editor and reviewer(s). You should upload this letter as a separate file labeled 'Response to Reviewers'.
- A marked-up copy of your manuscript that highlights changes made to the original version. You should upload this as a separate file labeled 'Revised Manuscript with Track Changes'.
- An unmarked version of your revised paper without tracked changes. You should upload this as a separate file labeled 'Manuscript'.

If you would like to make changes to your financial disclosure, please include your updated statement in your cover letter. Guidelines for resubmitting your figure files are available below the reviewer comments at the end of this letter.

If applicable, we recommend that you deposit your laboratory protocols in protocols.io to enhance the reproducibility of your results. Protocols.io assigns your protocol its own identifier (DOI) so that it can be cited independently in the future. For instructions see: https://journals.plos.org/plosone/s/submission-guidelines#loc-laboratory-protocols. Additionally, PLOS ONE offers an option for publishing peer-reviewed Lab Protocol articles, which describe protocols hosted on protocols.io. Read more information on sharing protocols at https://plos.org/protocols?utm_medium=editorial-email&utm_source=authorletters&utm_campaign=protocols.

As the corresponding author, your ORCID iD is verified in the submission system and will appear in the published article. PLOS supports the use of ORCID, and we encourage all coauthors to register for an ORCID iD and use it as well. Please encourage your coauthors to verify their ORCID iD within the submission system before final acceptance, as unverified ORCID iDs will not appear in the published article. *Only*  the individual author can complete the verification step; PLOS staff *cannot*  verify ORCID iDs on behalf of authors.

We look forward to receiving your revised manuscript.

Kind regards,

Stephen D. Ginsberg, Ph.D.

Section Editor

PLOS One

Journal requirements:

When submitting your revision, we need you to address these additional requirements.

1. Please ensure that your manuscript meets PLOS ONE's style requirements, including those for file naming. The PLOS ONE style templates can be found at

https://journals.plos.org/plosone/s/file?id=wjVg/PLOSOne_formatting_sample_main_body.pdf and

https://journals.plos.org/plosone/s/file?id=ba62/PLOSOne_formatting_sample_title_authors_affiliations.pdf

2. Thank you for stating the following financial disclosure:

“Supported by the Ministry of Science and Higher Education of the Russian Federation, (Agreement 075-10-2025-017 from 27.02.2025)

Please state what role the funders took in the study.  If the funders had no role, please state: "The funders had no role in study design, data collection and analysis, decision to publish, or preparation of the manuscript."

If this statement is not correct you must amend it as needed.

Please include this amended Role of Funder statement in your cover letter; we will change the online submission form on your behalf.

3. Thank you for stating the following in the Funding Section of your manuscript:

“Supported by the Ministry of Science and Higher Education of the Russian Federation, (Agreement 075-10-2025-017 from 27.02.2025)”

We note that you have provided funding information that is currently declared in your Funding Statement. However, funding information should not appear in the Acknowledgments section or other areas of your manuscript. We will only publish funding information present in the Funding Statement section of the online submission form.

Please remove any funding-related text from the manuscript and let us know how you would like to update your Funding Statement. Currently, your Funding Statement reads as follows:

“Supported by the Ministry of Science and Higher Education of the Russian Federation, (Agreement 075-10-2025-017 from 27.02.2025)

Please include your amended statements within your cover letter; we will change the online submission form on your behalf.

4. Please include captions for your Supporting Information files at the end of your manuscript, and update any in-text citations to match accordingly. Please see our Supporting Information guidelines for more information: http://journals.plos.org/plosone/s/supporting-information.

If the reviewer comments include a recommendation to cite specific previously published works, please review and evaluate these publications to determine whether they are relevant and should be cited. There is no requirement to cite these works unless the editor has indicated otherwise.

**Comments to the Author**

1. Is the manuscript technically sound, and do the data support the conclusions?

Reviewer #1: Partly

Reviewer #2: Yes

**********

2. Has the statistical analysis been performed appropriately and rigorously?

Reviewer #1: N/A

Reviewer #2: N/A

**********

3. Have the authors made all data underlying the findings in their manuscript fully available?

The PLOS Data policy

Reviewer #1: Yes

Reviewer #2: Yes

**********

4. Is the manuscript presented in an intelligible fashion and written in standard English?

Reviewer #1: Yes

Reviewer #2: No

**********

Reviewer #1: I agree with the authors that a review of rise-time processing is relevant to the field. Nevertheless, the quality of the written manuscript, more specifically the readability and clarity, should be improved. Please use tools (e.g., Grammarly or similar) to check the spelling and correct ill-formed sentences.

MAJOR

*) The article doesn't read easily. Sentences are poorly connected. Please, improve the introduction so that the reader is better guided to the neural processing of rise times and their link with speech processing. Some examples of what should be improved:

- L 45-46: The authors mention that processing of acoustical characteristics is linked to speech development. The second sentence is about disturbances in phonological processing; however, there is no link made between acoustic processing and phonological processing. Please better introduce how the processing of acoustics, specifically rise times, is linked to phonemes and phonological processing.

- L 57: "RT of formants affect speech perception", guide the reader on how RT affects speech perception.

- L 65: "discrimination of RT affects accuracy of linguistic representations". Which linguistic representation? How is this affected? Additionally, this statement should have a reference.

*) How are the queries determined. I feel that quite a few studies might be overlooked by restricting the queries. For example, why were meg studies not included? Why were terms such as auditory edges or acoustic boundaries not included?

MINOR

*) The resolution of the included images is too low. Please improve.

*) I think that some visual illustration would help the reader. For example, an overview figure with a short and long rise time sound, and an overview of the effect on the different components of the neural response.

*) correct spelling errors, among others:

L199: add which: "which were reviewed"

L234: Please add a comma between "onset brainstem response" and "offset brainstem response"

*) L221: "the latency of waves VI-VII first decreases (between 10µs and 0.5ms) and then gradually increases". This is vaguely formulated. Does the latency decrease and gradually increase when the rise time is varied between 10µs and 0.5ms, or is this a habituation effect whereby the latencies first decrease and subsequently increase based on the same rise time stimulus? Moreover, in the subsequent text, please either mention or provide an overview (in a small table) of how the rise time was varied in the referenced articles.

*) L235: "long-duration tone burst", is 8ms significantly longer than the cited preceding literature about the effect of RT on the ABR? What is the reason that the RT affects the offset response?

*) after each section (e.g.: early latency components), it would be good to provide a small summary. Additionally, if I'm not mistaken, most of the work was on adults. So it might be good to present the main results for adults, then specify the observed differences across populations.

*) L371: I'm not familiar with the T-complex. Is it the same Na peaks / neural responses you are referring to at the beginning of the section regarding middle latency components? How are the P0, Na, Pa, Nb, Pb components related to the well-known P1-N1-P2 components?

*) Regarding Difference Waves: How relevant is it to study these when the purely acoustic response to the stimulus with a deviant RT differs? Comparing different literature, what is the RT difference between standard and oddball stimuli? Would a larger difference imply a higher mismatch response?

*) L543: "individuals with dyslexia perform similarly to typical readers in speech in noise perception". But Van Hirtum et al. states: "In contrast, group differences were found for the speech-in-noise perception task (mean difference = .95 dB, SE = .25 dB, p < .001), indicating that SRTs were better for typical readers than for subjects with dyslexia."

*) Another limitation is that generally the number of included participants in the reviewed studies are rather low. This might also explain divergent effects.

*) It would be valuable to note that acoustic onsets are also investigated through continuous speech: see, among others, Rapid Transformation from Auditory to Linguistic Representations of Continuous Speech by Brodbeck et al. Perhaps the reader should be introduced to this kind of literature too, since the obtained effects are similar to the classical P1-N1-P2-complex.

Reviewer #2: See attached review. I have commented on the area of my expertise, but recommend a review by someone with greater auditory pathway EEG measures and understanding.

The typographical and grammatical errors are included in the attached Review.

**********

what does this mean?). If published, this will include your full peer review and any attached files.

If you choose “no”, your identity will remain anonymous but your review may still be made public.

**Do you want your identity to be public for this peer review?**  For information about this choice, including consent withdrawal, please see our Privacy Policy

Reviewer #1: No

Reviewer #2: No

[NOTE: If reviewer comments were submitted as an attachment file, they will be attached to this email and accessible via the submission site. Please log into your account, locate the manuscript record, and check for the action link "View Attachments". If this link does not appear, there are no attachment files.]

To ensure your figures meet our technical requirements, please review our figure guidelines: https://journals.plos.org/plosone/s/figures

You may also use PLOS’s free figure tool, NAAS, to help you prepare publication quality figures: https://journals.plos.org/plosone/s/figures#loc-tools-for-figure-preparation.

NAAS will assess whether your figures meet our technical requirements by comparing each figure against our figure specifications.

Attachment Submitted filename: 2026.7.21PLOS Review DBW.docx

# Author comment: Author response to Decision Letter 1

**DOI:** 10.1371/journal.pone.0345343.r002

14 Aug 2026

We're duplicating our responses to the editor and reviewers here. For convenience, a separate file with these responses, titled "Response to Reviewers," is attached to the submission.

Journal requirements:

When submitting your revision, we need you to address these additional requirements.

1. Please ensure that your manuscript meets PLOS ONE's style requirements, including those for file naming. The PLOS ONE style templates can be found at

https://journals.plos.org/plosone/s/file?id=wjVg/PLOSOne_formatting_sample_main_body.pdf and

https://journals.plos.org/plosone/s/file?id=ba62/PLOSOne_formatting_sample_title_authors_affiliations.pdf

Dear PLOS ONE Editorial Team,

Thank you for your email and for the opportunity to revise our manuscript (PONE-D-26-09613). We have carefully addressed each of the additional requirements outlined in your letter. Below we respond to each point in detail.

We have formatted the manuscript according to the PLOS ONE style templates.

2. Thank you for stating the following financial disclosure:

“Supported by the Ministry of Science and Higher Education of the Russian Federation, (Agreement 075-10-2025-017 from 27.02.2025)

Please state what role the funders took in the study. If the funders had no role, please state: "The funders had no role in study design, data collection and analysis, decision to publish, or preparation of the manuscript."

If this statement is not correct you must amend it as needed.

Please include this amended Role of Funder statement in your cover letter; we will change the online submission form on your behalf.

2. We confirm that the funders had no role in the study. The following amended Role of Funder statement has been included in our cover letter, as requested:

"Supported by the Ministry of Science and Higher Education of the Russian Federation, (Agreement 075-10-2025-017 from 27.02.2025). The funders had no role in study design, data collection and analysis, decision to publish, or preparation of the manuscript."

We have also updated the online submission form accordingly.

3. Thank you for stating the following in the Funding Section of your manuscript:

“Supported by the Ministry of Science and Higher Education of the Russian Federation, (Agreement 075-10-2025-017 from 27.02.2025)”

We note that you have provided funding information that is currently declared in your Funding Statement. However, funding information should not appear in the Acknowledgments section or other areas of your manuscript. We will only publish funding information present in the Funding Statement section of the online submission form.

Please remove any funding-related text from the manuscript and let us know how you would like to update your Funding Statement. Currently, your Funding Statement reads as follows:

“Supported by the Ministry of Science and Higher Education of the Russian Federation, (Agreement 075-10-2025-017 from 27.02.2025)

Please include your amended statements within your cover letter; we will change the online submission form on your behalf.

3. We confirm that all funding-related text has been removed from the manuscript. No funding information appears in the Acknowledgments or any other section of the manuscript.

4. Please include captions for your Supporting Information files at the end of your manuscript, and update any in-text citations to match accordingly. Please see our Supporting Information guidelines for more information: http://journals.plos.org/plosone/s/supporting-information.

4. We have added a dedicated "Supporting Information" section at the end of the manuscript, with captions for all supporting files:

S1 Table. OHAT risk of bias description and table.

S2 Table. Summary details of the included studies. The studies are divided into groups according to the experimental paradigm used, and organized within each group in ascending order of year.

S3 Table. PRISMA checklist.

All in-text citations have been updated to match these labels (e.g., "S1 Table", "S2 Table").

We believe we have now fully addressed all editorial requirements. Please let us know if any further adjustments are needed.

If the reviewer comments include a recommendation to cite specific previously published works, please review and evaluate these publications to determine whether they are relevant and should be cited. There is no requirement to cite these works unless the editor has indicated otherwise.

Reviewer comments:

Reviewer #1: I agree with the authors that a review of rise-time processing is relevant to the field. Nevertheless, the quality of the written manuscript, more specifically the readability and clarity, should be improved. Please use tools (e.g., Grammarly or similar) to check the spelling and correct ill-formed sentences.

Dear Reviewer,

We would like to thank you for your thorough and constructive comments on our manuscript. We have carefully addressed each of the points raised and believe the revised version is substantially improved. Below we provide our point‑by‑point responses. All changes in the manuscript have been marked using track changes.

In response to the comment on language and readability, we have carefully revised the entire manuscript. We restructured awkward sentences, corrected grammatical and spelling errors, and improved the overall flow. To assist with this process, we used several language-editing tools, including the Google Docs extension 'GPT — Grammar and Spelling Check', Paperpal, and the AI‑powered grammar and spelling check powered by Gemini, alongside manual revision. We believe the revised version now reads more smoothly and is better organised for the reader.

MAJOR

*) The article doesn't read easily. Sentences are poorly connected. Please, improve the introduction so that the reader is better guided to the neural processing of rise times and their link with speech processing. Some examples of what should be improved:

We have substantially revised the introduction to establish a clear and logical link between acoustic processing of rise time, phonological representations, and speech development.

- L 45-46: The authors mention that processing of acoustical characteristics is linked to speech development. The second sentence is about disturbances in phonological processing; however, there is no link made between acoustic processing and phonological processing. Please better introduce how the processing of acoustics, specifically rise times, is linked to phonemes and phonological processing.

We have added an explanation of how rise time cues contribute to parsing the acoustic stream into discrete phonological units. We now provide concrete examples of phonemic contrasts (/b/ vs /w/ and /tʃ/ vs /ʃ/) to illustrate how RT shapes phonetic categorisation. (L26 - 36)

- L 57: "RT of formants affect speech perception", guide the reader on how RT affects speech perception.

We have clarified how rise time affects speech perception:

L 26-39: “In natural speech, RT cues are critical for parsing the continuous acoustic stream into discrete phonological units. For example, the distinction between the stop consonant /b/ and the glide /w/ relies partly on the rate of amplitude rise at sound onset: rapid amplitude increase produces abrupt stop/click, whereas slower rise is perceived as a glide [3]. Similarly, the distinction between the voiceless affricate /tʃ/ and the fricative /ʃ/ also involves differences in the amplitude envelope's rise characteristics, although the perceptual boundary between these categories is not fixed but shifts depending on the stimulus context [4]. Thus, amplitude RT contributes directly to phonetic categorization across multiple types of consonant contrasts. It should be noted that RT is sometimes distinguished into amplitude RT and formant RT – the rate at which formant frequencies reach their target values; the two are often discussed together, but the present review focuses on the amplitude rise time specifically.

Accurate RT perception is thought to support the formation of stable phonological representations, which in turn are essential for mapping sounds to letters during reading acquisition [1,2]”

- L 65: "discrimination of RT affects accuracy of linguistic representations". Which linguistic representation? How is this affected? Additionally, this statement should have a reference.

We have replaced the vague term “linguistic representations” with “phonological representations” and added an explicit account of the mechanism linking rise time discrimination to phonological category stability. The statement is now supported by an appropriate reference (Goswami, 2002).

(For the full revised introduction, please see lines 19–60)

*) How are the queries determined. I feel that quite a few studies might be overlooked by restricting the queries. For example, why were meg studies not included? Why were terms such as auditory edges or acoustic boundaries not included?

We have added a clear justification in the Methods section (Search strategy, lines 76–87):

“Thus, the search strategy was focused on EEG-based studies, as the majority of systematic investigations of rise-time processing – where rise time is varied as an independent variable – have been conducted using this method. While a small number of magnetoencephalography (MEG) studies have examined rise-time-related phenomena (e.g., sound detection of slowly rising intensity, perceptual asymmetry for ramped vs. damped sounds), they address different research questions and do not systematically vary rise time as a phonetic/acoustic parameter; therefore, they were not included in order to maintain the coherence of the reviewed evidence. We used the specific term "rise time" to target studies directly investigating this acoustic parameter. Terms such as "auditory edges" or "acoustic boundaries" were not included, as they typically refer to studies on general auditory scene segmentation and spectrotemporal boundary detection, which are conceptually distinct from rise time as a parameter of speech signals.”

MINOR

*) The resolution of the included images is too low. Please improve.

We have replaced all figures with high‑resolution versions (>300 dpi, TIFF and EPS format) to meet PLOS ONE requirements.

*) I think that some visual illustration would help the reader. For example, an overview figure with a short and long rise time sound, and an overview of the effect on the different components of the neural response.

We agree that a schematic illustration would be helpful. We have prepared an overview figure showing a short vs long rise time sound and its typical effects on the amplitude and latency of the main ERP components. This figure is included in the revised manuscript (Fig. 3A).

*) correct spelling errors, among others:

We have corrected the spelling and grammatical issues noted.

L199: add which: "which were reviewed"

We have revised the sentence to improve clarity. The original wording was awkward; we have rephrased it: “Some studies described more than one category of EEG-response and were therefore reviewed in several sections. ” (now L144-145)

L234: Please add a comma between "onset brainstem response" and "offset brainstem response"

The section about the offset brainstem response has been rewritten to improve grammar and readability:

“One of the articles reported an offset brainstem response that was morphologically similar to the onset brainstem response. This offset response occurred within 8 ms after stimulus offset for tone bursts exceeding 8 ms in duration [21]. Offset ABR was even more affected by stimulus RT than onset ABR. In one experiment, where RT was varied across four values (0.5, 1.0, 2.5, and 5.0 ms), both offset and onset wave V amplitude decreased and latency increased with longer RT. However, due to an increasing number of missing responses at longer RTs, only the 0.5 and 1.0 ms conditions were included in the statistical analysis; the 2.5 and 5.0 ms conditions were excluded [21]. In the other experiment, where RT was varied across two values (0.5 and 5 ms), significant effects were observed only for the offset ABR: offset wave V latency increased for both 500 Hz and 2000 Hz stimuli, and offset amplitude decreased for 2000 Hz stimuli as rise time increased from 0.5 to 5.0 ms [21].” (now L176-186).

*) L221: "the latency of waves VI-VII first decreases (between 10µs and 0.5ms) and then gradually increases". This is vaguely formulated. Does the latency decrease and gradually increase when the rise time is varied between 10µs and 0.5ms, or is this a habituation effect whereby the latencies first decrease and subsequently increase based on the same rise time stimulus? Moreover, in the subsequent text, please either mention or provide an overview (in a small table) of how the rise time was varied in the referenced articles.

We have rewritten this part to clarify that the latency changes reflect the effect of varying rise time across experimental conditions, not a habituation effect. We specify the rise time values used in all studies in Table S1 (column “RT”), so, we have added a reference to Table S2 at the end of the ABR section, which provides a comprehensive overview of all RT conditions used in the reviewed ABR studies (lines 161–167 and 208).

*) L235: "long-duration tone burst", is 8ms significantly longer than the cited preceding literature about the effect of RT on the ABR? What is the reason that the RT affects the offset response?

We have restructured this paragraph to clarify that “long‑duration” refers to the total duration of the tone burst (>8 ms), not to its rise time, and that the offset ABR was more sensitive to RT manipulation than the onset ABR, consistent with evidence that onset and offset ABRs are generated by distinct mechanisms, with the offset response arising from more apical regions of the cochlea:

“One of the articles reported an offset brainstem response that was morphologically similar to the onset brainstem response. This offset response occurred within 8 ms after stimulus offset for tone bursts exceeding 8 ms in duration [21]. Offset ABR was even more affected by stimulus RT than onset ABR. In one experiment, where RT was varied across four values (0.5, 1.0, 2.5, and 5.0 ms), both offset and onset wave V amplitude decreased and latency increased with longer RT. However, due to an increasing number of missing responses at longer RTs, only the 0.5 and 1.0 ms conditions were included in the statistical analysis; the 2.5 and 5.0 ms conditions were excluded [21]. In the other experiment, where RT was varied across two values (0.5 and 5 ms), significant effects were observed only for the offset ABR: offset wave V latency increased for both 500 Hz and 2000 Hz stimuli, and offset amplitude decreased for 2000 Hz stimuli as rise time increased from 0.5 to 5.0 ms [21]. Van Campen et al. proposed that the offset ABR is generated by a more apical region of the cochlea, which would explain its greater sensitivity to RT and its stronger elicitation by low-frequency stimuli. “ (now L176-188)

*) after each section (e.g.: early latency components), it would be good to provide a small summary. Additionally, if I'm not mistaken, most of the work was on adults. So it might be good to present the main results for adults, then specify the observed differences across populations.

We have added a brief summary at the end of each main section that included more than two studies: ABR (L204-208), P1 (L243-245), N1 (L276-279), P2 (L314-317), T‑complex (L332-335), N2 (L350-351), and difference waves (L480–490). Each summary first presents the main findings in adults and then highlights any observed differences across populations (children, dyslexia) or notes where such data are lacking.

*) L371: I'm not familiar with the T-complex. Is it the same Na peaks / neural responses you are referring to at the beginning of the section regarding middle latency components? How are the P0, Na, Pa, Nb, Pb components related to the well-known P1-N1-P2 components?

We have expanded the T‑complex section (lines 320–324) to avoid confusion:

“The T-complex captures N1 subcomponents recorded over temporal electrodes (e.g., T7/T8): Na (N1a, 75–95 ms), Ta (N1b, 100–115 ms), and Tb (N1c, 130–170 ms) [43]. This should not be confused with the earlier MLR Na wave (~20–30 ms). The T-complex reflects activity in lateral superior temporal gyrus and planum temporale [43,44]. Two of the studies included in the review examined T-complex RT. “

*) Regarding Difference Waves: How relevant is it to study these when the purely acoustic response to the stimulus with a deviant RT differs? Comparing different literature, what is the RT difference between standard and oddball stimuli? Would a larger difference imply a higher mismatch response?

We have added a summary to address this issue to the “Difference Waves” section (lines 486–490):

“Regarding the magnitude of the standard–deviant RT contrast, the values ranged widely across reviewed studies from 22 [56] to 278 ms [52]. Notable, only Choisdealbha et al. [52] systematically varied the RT difference in five steps (161.1–292.7 ms), and demonstrated that larger RT differences elicited larger and more positive mismatch responses.”

We also added the following text to the “Limitations” section (L798-803):

“While the oddball paradigm allows assessing the neurophysiological response to sound differences, it has several drawbacks, such as increased duration of stimulation, a low signal-to-noise ratio, ambiguity in result interpretation, and difficulties in disentangling neural responses to RT changes from responses to other acoustic differences between standard and deviant stimuli [50,76]. Thus, recent neurophysiological studies on RT can be largely supplemented by the analysis of main ERP components in response to repetitive stimulation.”

*) L543: "individuals with dyslexia perform similarly to typical readers in speech in noise perception". But Van Hirtum et al. states: "In contrast, group differences were found for the speech-in-noise perception task (mean difference = .95 dB, SE = .25 dB, p < .001), indicating that SRTs were better for typical readers than for subjects with dyslexia."

We have corrected the text (lines 500–502) to state that in Van Hirtum et al. (2019), typical readers performed significantly better than participants with dyslexia on the speech‑in‑noise perception task, while performance on reaction time and intensity discrimination was similar between groups.

*) Another limitation is that generally the number of included participants in the reviewed studies are rather low. This might also explain divergent effects.

We have added a note on small sample sizes to the Limitations section (L859–861), acknowledging that many reviewed studies (particularly older ABR and middle‑latency studies) have N < 15, which may limit generalisability and statistical power and could contribute to divergent results across studies.

*) It would be valuable to note that acoustic onsets are also investigated through continuous speech: see, among others, Rapid Transformation from Auditory to Linguistic Representations of Continuous Speech by Brodbeck et al. Perhaps the reader should be introduced to this kind of literature too, since the obtained effects are similar to the classical P1-N1-P2-complex.

We agree that this is an important emerging area. We have added a new paragraph to the “Directions for further research” section (L805–813), discussing the use of temporal response function (TRF) modelling to investigate acoustic onsets in continuous speech, referencing Brodbeck et al. (2018), Power et al. (2016), and Borges et al. (2025). We also highlight the potential application of these approaches to clinical populations (e.g., dyslexia).

We are grateful for the constructive feedback and hope that the revised version now fully addresses all concerns.

Reviewer #2: See attached review. I have commented on the area of my expertise, but recommend a review by someone with greater auditory pathway EEG measures and understanding.

The typographical and grammatical errors are included in the attached Review.

Reviewer: D. Bradley Welling, MD, PhD

Scope of This Review: I was asked to review this manuscript to offer a clinical

perspective on its relevance to patient populations within my area of practice. I want to

note at the outset that my expertise is clinical rather than in EEG signal-processing

methodology, event-related potential paradigm design, or psychophysical experimental

methods — which make up the majority of this manuscript's content. My comments

below are accordingly limited to (1) clinical relevance and accessibility for a clinical

readership, and (2) points of language clarity that affected my ability, as a non-specialist

reader, to follow specific sections. I would defer questions of methodological rigor,

statistical approach, and completeness of the systematic review process to a reviewer

with more direct expertise in EEG/ERP methodology.

Dear Dr. Welling,

We thank you very much for your thoughtful and constructive review. We greatly appreciate that you took the time to provide a clinical perspective on our manuscript, and we have taken your comments very seriously. Below we respond to each of your points.

General Comments

● Clinical relevance and accessibility. Although the review touches on populations of

clinical interest (dyslexia, ASD, speech-language disorders), the manuscript remains

oriented primarily toward basic psychophysics and EEG methodology. A clinical reader

would benefit from a more explicit paragraph connecting rise-time processing markers

to diagnostic or therapeutic relevance.

We agree that the manuscript was initially oriented primarily toward basic psychophysics and methodology. In response, we have added an explicit paragraph in the Discussion (lines 814–826) that directly connects RT processing markers to diagnostic and therapeutic relevance. The paragraph now reads:

“The reviewed findings suggest potential clinical relevance, but also highlight the current limitations of the evidence. EEG/ERP markers of RT sensitivity, such as reduced N1–P2 amplitude modulation, absent or atypical MMN/MMR responses, and altered T‑complex activity, have been reported in individuals with dyslexia, but the findings are inconsistent across studies. Such markers could be used for early identification of children at risk for reading impairments or serve as outcome measures for auditory training interventions. For example, reduced MMN/MMR to RT deviants in pre‑reading children has been proposed as a potential early risk marker for dyslexia [85], and prolonged P1 latency or attenuated N1c amplitude may index atypical cortical development [30]. However, the translational value of these findings is currently limited by methodological heterogeneity, small sample sizes, and the lack of standardised protocols. At present, no single EEG/ERP marker of RT processing has been consistently validated across studies, and the available data do not support clinical application. Future research should prioritise replication in large, well‑characterised cohorts and the establishment of normative data before any clinical use can be considered.”

We believe this addition makes the clinical relevance more explicit and accessible to a clinical readership.

● Electrode placement schematic. I would suggest adding a figure illustrating typical

electrode placement/montage for the ERP components discussed (ABR, MMN, N1P2,

etc.). This would help readers less familiar with EEG methodology follow the anatomical

and temporal correspondence of the components being compared. (в саммари)

We agree that a schematic illustrating typical electrode placement for the ERP components discussed (ABR, MMN, N1/P2, etc.) would greatly help readers less familiar with EEG methodology. We have added this information to a schematic figure (Fig. 3B) that illustrates the ERP components alongside their typical recording sites. We hope this improves accessibility for non-specialist readers.

● Abbreviation definitions. Given the density of abbreviations (ABR, ASSR, ERP, MMN,

MMR, LDN, RT, FT, and clinical terms such as ASD), I recommend a full pass to confirm

every abbreviation is defined at first use in the body text, not only in the supplementary

abbreviation table (see line 924 below).

We have carefully reviewed the entire manuscript and confirmed that every abbreviation is defined at first use in the body text. In particular, we have verified all abbreviations (ABR, ASSR, ERP, MMN, MMR, LDN, RT, FT, ART, FRT, ISI, etc.) are spelled out upon first appearance.

● Length and structure. The manuscript is quite long. The authors may wish to consider

whether some of the more granular study-by-study parameter comparisons (e.g., the

results subsections cataloguing RTdifference ranges across individual studies) could be

moved to supplementary tables, which might improve readability without loss of content.

We agree with the concern regarding the length of the manuscript. In response, we have substantially revised the text: we have shortened and reformulated large sections to improve conciseness while preserving the essential information. In addition, we have moved the detailed study‑by‑study parameter comparisons to Supplementary Table S2, and we have created a new Figure 3, providing a more accessible overview of the key findings. We believe these changes have made the manuscript clearer and more reader‑friendly without compromising the completeness of the systematic review.

Specific Comments

Line 234–235 The sentence is difficult to parse: “One of the articles reported

morphologically similar to onset brainstem response offset brainstem response which

occurs by long-duration tone burst (>8 ms) within 8 ms after stimulus offset [22].”

The sentence needs restructuring. Suggested revision: “One of the articles reported an

offset brainstem response that was morphologically similar to the onset brainstem

response and occurred within 8 ms of stimulus offset for long-duration tone bursts (>8

ms) [22].”

Lines 176–178: The sentence has been restructured as follows:

“One of the articles reported an offset brainstem response that was morphologically similar to the onset brainstem response. This offset response occurred within 8 ms after stimulus offset for tone bursts exceeding 8 ms in duration. [23].“

Lines 695–697 “…the auditory system has fewer activation, leading to poorer

discrimination.” ‘Fewer’ does not grammatically modify the uncountable noun

‘activation.’ Suggest ‘less activation’ or, to align with the wording in the following

sentence, ‘fewer activated auditory nerve fibers.’

Lines 626: We have corrected "fewer activation" to "less activation". (now line 665)

Line 707 “…an intensity condition of 15 was used, at this condition response was above

noise level for only one participant…” This is a comma splice; the sentence should be

split into two after ‘used.’ Separately, please clarify the units for ‘intensity condition of

15’ (presumably dB SPL or dB HL) — as written it is ambiguous.

Line 634-635: We have rewritten the sentence and clarified the units: "At 15 dB SPL the response was reliably detectable for only one participant in the 3-ms RT condition, and for only two participants in the 30-ms RT condition. "

Lines 737–739 Subject–verb agreement: “…the components modulation were

demonstrated for all used contrasts. The range of RT differences, which showed

significant results for late latency (components was from 4.99 ms to 500 ms…”

‘Components was’ should read ‘components were.’

Line 660: We have corrected the subject–verb agreement and reformulated the sentence for better readability: "The late latency components were sensitive to RT from 5 to 500 ms."

Lines 752–754 “…researchers typically present target sounds gradually logarithmically

ranging from 15 ms (the standard) to longer durations.” This phrase is unclear to me as

a non-specialist reader. Please clarify whether this refers to stimulus values spaced

logarithmically across a study/staircase procedure, or to some other aspect of stimulus

presentation.

Lines 671–675: We have substantially revised the text and clarified that this refers to stimulus values spaced logarithmically across a staircase procedure: “A baseline of 15 ms is a standard in cortical RT studies [27,31,48,49,62]. This value avoids spectral splatter (which occurs with very short RTs) while maintaining temporal precision [68], and aligns with critical RTs in the speech envelope that impact intelligibility [69]. In behavioral tasks, RTs are typically spaced logarithmically from this baseline in staircase procedures [63].“

Line 924 “…with ASD [81].” The abbreviation ‘ASD’ is used here and repeated at lines

926, 928, and 929, but is never spelled out in the body text — it appears written out only

within reference titles in the bibliography. Recommend defining ‘Autism Spectrum

Disorder (ASD)’ at first use.

We have removed the brief reference to ASD from the clinical relevance discussion, as suggested further.

Comment

Would any of the parameters summarized in this review be of use with auditory cortical

stimulation with AI algorithms in the future? Auditory brainstem implant (ABI) users

experience a wide range of open-set speech understanding, with many users unable to

benefit substantially. Could future studies utilize the EEG response to ABI signals to

better understand the way the auditory cortex is processing these signals and be useful

in designing more advanced stimulation parameters similar to the information gleaned

from Moberly et al.

I found the information on differences in EEG response in dyslexia interesting and

would welcome more information on related topics. The brief reference to Autism

Spectrum Disorders should be described in more detail or omitted.

We thank you for this forward-looking question. We have added a paragraph in the Directions for further research section (lines 768–781) addressing the potential relevance of EEG measures of RT discrimination for auditory brainstem and midbrain implants. We now discuss the work of Moberly et al. (2016) in cochlear implant users, and note that similar approaches could be used to characterise cortical processing of implant-generated signals and to guide stimulation parameter optimisation. We also reference Nager et al. (2008), who recorded MMN in response to RT deviants in a patient with an auditory midbrain implant. We have also included a separate paragraph (lines 827–843) on the potential integration of RT-related EEG/ERP markers into machine learning frameworks for early screening, referencing recent work on EEG‑based classification of dyslexia and other neurodevelopmental conditions.

We also have removed the brief reference to ASD from the clinical relevance discussion, as suggested. We agree that without dedicated studies on RT processing in ASD, it is better not to include it.

Recommendation: Recognizing that my review is limited to language clarity and clinical-

relevance considerations rather than methodological or statistical evaluation, I

recommend minor revision, contingent on: (1) a language-editing pass addressing the

issues noted above (and likely others outside my ability to catch, given the technical

density of the material); (2) clarification of the ambiguous methodological language

flagged above; (3) consideration of an electrode-placement schematic; and (4)

confirmation from a reviewer with direct EEG/ERP methodological expertise regarding

the completeness and rigor of the systematic review process itself, which falls outside

my area of qualification to assess.

We have carefully revised the entire manuscript for clarity and flow. We restructured awkward sentences, corrected grammatical and spelling errors, and improved the overall flow. To assist with this process, we used several language-editing tools, including the Google Docs extension "GPT – Grammar and Spelling Check", Paperpal, and the AI‑powered grammar and spelling check powered by Gemini, alongside manual revision. We believe the revised version now reads more smoothly and is better organised for the reader. We are grateful for your constructive feedback and hope that the revised version now fully addresses all your concerns.

Attachment Submitted filename: Response to reviewers.docx

# Peer review: Decision Letter 1

**DOI:** 10.1371/journal.pone.0345343.r003

**Authors:** Stephen Ginsberg

13 Sep 2026

<p>EEG correlates of auditory rise time processing: A systematic review

PONE-D-26-09613R1

Dear Dr. Manasevich,

We’re pleased to inform you that your manuscript has been judged scientifically suitable for publication and will be formally accepted for publication once it meets all outstanding technical requirements.

Within one week, you’ll receive an e-mail detailing the required amendments. When these have been addressed, you’ll receive a formal acceptance letter and your manuscript will be scheduled for publication.

An invoice will be generated when your article is formally accepted. Please note, if your institution has a publishing partnership with PLOS and your article meets the relevant criteria, all or part of your publication costs will be covered. Please make sure your user information is up-to-date by logging into Editorial Manager at Editorial Manager® and clicking the ‘Update My Information' link at the top of the page. For questions related to billing, please contact billing support.

If your institution or institutions have a press office, please notify them about your upcoming paper to help maximize its impact. If they’ll be preparing press materials, please inform our press team as soon as possible -- no later than 48 hours after receiving the formal acceptance. Your manuscript will remain under strict press embargo until 2 pm Eastern Time on the date of publication. For more information, please contact onepress@plos.org.

Kind regards,

Stephen D. Ginsberg, Ph.D.

Section Editor

PLOS One

**Comments to the Author**

Reviewer #2: All comments have been addressed

**********

2. Is the manuscript technically sound, and do the data support the conclusions?

Reviewer #2: Yes

**********

3. Has the statistical analysis been performed appropriately and rigorously?

Reviewer #2: I Don't Know

**********

4. Have the authors made all data underlying the findings in their manuscript fully available?

The PLOS Data policy

Reviewer #2: Yes

**********

5. Is the manuscript presented in an intelligible fashion and written in standard English?

Reviewer #2: Yes

**********

Reviewer #2: My queries have been answered. I have no further questions or comments for the authors. Thank you for a thorough response.

**********

what does this mean?). If published, this will include your full peer review and any attached files.

If you choose “no”, your identity will remain anonymous but your review may still be made public.

**Do you want your identity to be public for this peer review?**  For information about this choice, including consent withdrawal, please see our Privacy Policy

Reviewer #2: No

# Editor report: Acceptance letter

**DOI:** 10.1371/journal.pone.0345343.r004

**Authors:** Stephen Ginsberg

PONE-D-26-09613R1

PLOS One

Dear Dr. Manasevich,

I'm pleased to inform you that your manuscript has been deemed suitable for publication in PLOS One. Congratulations! Your manuscript is now being handed over to our production team.

At this stage, our production department will prepare your paper for publication. This includes ensuring the following:

* All references, tables, and figures are properly cited

* All relevant supporting information is included in the manuscript submission,

* There are no issues that prevent the paper from being properly typeset

You will receive further instructions from the production team, including instructions on how to review your proof when it is ready. Please keep in mind that we are working through a large volume of accepted articles, so please give us a few days to review your paper and let you know the next and final steps.

Lastly, if your institution or institutions have a press office, please let them know about your upcoming paper now to help maximize its impact. If they'll be preparing press materials, please inform our press team within the next 48 hours. Your manuscript will remain under strict press embargo until 2 pm Eastern Time on the date of publication. For more information, please contact onepress@plos.org.

You will receive an invoice from PLOS for your publication fee after your manuscript has reached the completed accept phase. If you receive an email requesting payment before acceptance or for any other service, this may be a phishing scheme. Learn how to identify phishing emails and protect your accounts at https://explore.plos.org/phishing.

If we can help with anything else, please email us at customercare@plos.org.

Thank you for submitting your work to PLOS One and supporting open access.

Kind regards,

PLOS One Editorial Office Staff

on behalf of

Dr. Stephen D. Ginsberg

Section Editor

PLOS One
